# Skill-driven recommendations for job transition pathways

**DOI:** 10.1371/journal.pone.0254722

**Published:** 2021-08-04

**Authors:** Nikolas Dawson, Mary-Anne Williams, Marian-Andrei Rizoiu

**Affiliations:** 1 Centre of Artificial Intelligence, University of Technology Sydney, Sydney, Australia; 2 Business School, University of New South Wales, Sydney, Australia; 3 Data Science Institute, University of Technology Sydney, Sydney, Australia; Xiamen University, CHINA

## Abstract

Job security can never be taken for granted, especially in times of rapid, widespread and unexpected social and economic change. These changes can force workers to transition to new jobs. This may be because new technologies emerge or production is moved abroad. Perhaps it is a global crisis, such as COVID-19, which shutters industries and displaces labor *en masse*. Regardless of the impetus, people are faced with the challenge of moving between jobs to find new work. Successful transitions typically occur when workers leverage their existing skills in the new occupation. Here, we propose a novel method to measure the similarity between occupations using their underlying skills. We then build a recommender system for identifying optimal transition pathways between occupations using job advertisements (ads) data and a longitudinal household survey. Our results show that not only can we accurately predict occupational transitions (Accuracy = 76%), but we account for the asymmetric difficulties of moving between jobs (it is easier to move in one direction than the other). We also build an early warning indicator for new technology adoption (showcasing Artificial Intelligence), a major driver of rising job transitions. By using real-time data, our systems can respond to labor demand shifts as they occur (such as those caused by COVID-19). They can be leveraged by policy-makers, educators, and job seekers who are forced to confront the often distressing challenges of finding new jobs.

## Introduction

In March 2020, COVID-19 caused entire industries to shutter as governments scrambled to ‘flatten the curve’. Jobs were lost or subject to an indefinite hiatus; firms went into ‘hibernation’ to wait out the depressed demand; and governments exercised wartime measures of labor redeployment and wage subsidies of unprecedented scale. All in a matter of weeks.

Labor market shocks, such as those caused by COVID-19, force workers to abruptly transition between jobs. Crises, however, are not the only cause of large-scale job transitions. Structural shifts in labor demand are another major obstacle [1], but usually unfold more gradually. Indeed, technological advances were expected to cause the next wave of major labor market disruptions [2, 3]. The ‘future of work’ was to be defined by technologies like Artificial Intelligence (AI); technologies that would automate and augment workers, but at the same time transform the requirements of jobs and the demand for labor *en masse* [4–6].

Despite the impetus, many workers need to transition between jobs. In some countries, such as Australia, labor displacement has increased over the past two decades with relatively high levels of job transitions [7]; a situation exacerbated by COVID-19 [8]. While job turnover is not innately negative and can be a signal of labor market dynamism, it does depend on how efficiently workers transition back into the workforce. Transitioning from one job to another can be difficult or unfeasible when the skills gap is too large [9]. Successful transitions typically involve workers leveraging their existing skills and acquiring new skills to meet the demands of the target occupation [10, 11]. Therefore, transitioning workers successfully at scale requires maximizing the similarity between workers’ current skills and their target jobs. Skills, knowledge areas, and capabilities enable workers to achieve tasks required by jobs [12]. We refer to these aspects of human capital as ‘skills’ throughout this research, and we characterize labor market entities (individual jobs, standardized occupations, industries, etc.) as *sets of skills*.

Here, we propose a novel method to measure the distance between sets of skills from more than 8 Million real-time job advertisements (ads) in Australia from 2012–2020. We call this data-driven methodology Skills Space. The Skills Space method enables us to measure the distance between any defined skill sets based on distances at the individual skill level. When two skill sets are highly similar (for example, two occupations), the skills gap is narrow, and the barriers to transitioning from one to the other are low. Drawing from previous work [9–11, 13], we construct a unique *Job Transitions Recommender System* that incorporates the skill set distance measures together with other labor market data from job ads and employment statistics. This allows us to account for a wealth of labor market variables from multiple sources. The outputs of the recommender system accurately predict transitions between occupations (Accuracy = 76%) and are validated against a dataset of occupational transitions from a longitudinal household survey [14]. While previous studies have analyzed job transitions using the same or similar job ads data [15–18], they have not accounted for the asymmetries between jobs (please refer to the S1 File for a detailed review of the related literature). Our system accounts for the asymmetries between occupations (it is easier to move in one direction than the other), leverages real-time job ads data at the granular skills-level, and accurately recommends occupations and skills that can assist workers looking to transition between jobs based on their personalized skills set. We further demonstrate the flexibility of the Skills Space method by constructing a leading indicator of Artificial Intelligence (AI) adoption within Australian industries. In our applications of Skills Space, we are able to both *recommend* transition pathways to workers based on their personalized skill sets and *detect* emerging AI disruption that could accelerate job transitions.

## Materials and methods

### Datasets and ground-truth

#### Job ads data

This research draws on 8,002,780 online job ads in Australia from 2012–01-01 to 2020–04-30, courtesy of Burning Glass Technologies (BGT). This dataset provides unique insights into the evolving labor demands of Australia. It also covers the early periods of the COVID-19 crisis when Australian governments closed ‘non-essential’ services [19]. To construct this dataset, BGT has systematically collected job ads via web-scraping. This process removes duplicates of job ads posted across multiple job boards or job ads re-posted in short time-frames. They also parse the unstructured job description text through their proprietary systems that extract key features from the advertised job. These features include location, employer, salary, education requirements, experience demands, occupational class, industry classifications, among others. Importantly for this research, the skill requirements have also been extracted (>11,000 unique skills). Here, BGT adopt a broad description of ‘skills’ to include skills, knowledge, abilities, and tools & technologies. This is slightly different to the more commonly used skills data from O*NET, which defines skills as a series of developed capacities that are categorized into different competencies [20]. There are two major advantages of using BGT job ads data over O*NET skills data: (1) more granular ‘skills’ data and (2) longitudinal (when used historically) and near-real-time skills data in specific locations. The latter point is particularly important when building a real-time job transitions recommender system to navigate labor crises as they unfold.

#### Employment statistics

The employment data used for this research is drawn from the ‘Quarterly Detailed Labor Force’ statistics by the Australian Bureau of Statistics (ABS) [21]. These data represent labor supply features for the 4-digit occupations in the *Job Transitions Recommender System* and include measures of employment levels and hours worked per occupation.

#### Occupational transitions ground-truth

The Household, Income and Labour Dynamics in Australia (HILDA) Survey is a nationally representative longitudinal panel study of Australian households that commenced in 2001 [14]. It has three main areas of interest: income, labor, and family dynamics. The HILDA survey is in its 18th consecutive year, with the latest available data available from 2018.

Included within the HILDA are data on occupational history and movements of anonymized respondents. We use this data to identify when respondents have changed jobs from one year to another. The occupations are recorded at the 4-digit level from the Australian and New Zealand Standard Classification of Occupations (ANZSCO). This shows the occupation of the previous year and the current year. We use this longitudinal dataset as the ground truth for validating Skills Space. As the job ads dataset used for this research begins at 2012, we isolate the observations of occupational transitions from 2012 to 2018 (the latest available year). This results in a sample of 2,999 occupational transitions in Australia.

### Measuring skill similarity

To measure the distance between occupations (or other skill groups), we first measure the pairwise distance between individual skills (6,981 skills in 2018) in jobs ads for each calendar year from 2012–2020. Intuitively, two skills are similar when they are simultaneously important for the same set of job ads. We measure the importance of a skill in a job ad using an established measure called ‘Revealed Comparative Advantage’ (*RCA*— Eq (1)) that has been applied across a range of disciplines, such as trade economics [22, 23], identifying key industries in nations [24], detecting the labor polarization of workplace skills [25], and adaptively selecting occupations according to their underlying skill demands [26]. *RCA* normalizes the total share of demand for a given skill across all job ads. We then calculate the pairwise skill similarity between each skill using Eq (2) as implemented by Alabdulkareem et al. [25] and again by Dawson et al. [26]. These individual skill distances form the basis for measuring the distance between sets of skills.

To measure the distance between every skill for each year in the dataset, we start by removing extremely rare skills. Here, we select skills with a posting frequency count > = 5, which represent ∼75% of all skills (see S1 File for more details). Let S be the set of all skills and J be the set of all job ads in our dataset. We measure the similarity between two individual skills *s*_1_ and *s*_2_ ($s_{1},s_{2}\in S$) using a methodology proposed by Alabdulkareem et al. [25]. First, we use the *Revealed Comparative Advantage* (RCA) to measure the importance of a skill *s* for a particular job ad *j*:

$$\begin{array}{c} RCA\left(j,s\right)=\frac{x\left(j,s\right)/\sum_{s^{\prime }\in S}x\left(j,s^{\prime }\right)}{\sum_{j^{\prime }\in J}x\left(j^{\prime },s\right)/\sum_{j^{\prime }\in J,s^{\prime }\in S}x\left(j^{\prime },s^{\prime }\right)} \end{array}$$ (1)

where *x*(*j*, *s*) = 1 when the skill *s* is required for job *j*, and *x*(*j*, *s*) = 0 otherwise; $RCA\left(j,s\right)\in [0,\sum_{j^{\prime }\in J,s^{\prime }\in S}x\left(j^{\prime },s^{\prime }\right)],\forall j,s$, and the higher *RCA*(*j*, *s*) the higher is the comparative advantage that *s* is considered to have for *j*. Visibly, *RCA*(*j*, *s*) decreases when the skill *s* is more ubiquitous (i.e. when $\sum_{j^{\prime }\in J}x\left(j^{\prime },s\right)$ increases), or when many other skills are required for the job *j* (i.e. when $\sum_{s^{\prime }\in S}x\left(j,s^{\prime }\right)$ increases). Next, we measure the similarity between two skills based on the likelihood that they are both effectively used in the same job ads. Formally:

$$\begin{array}{c} \theta \left(s_{1},s_{2}\right)=\frac{\sum_{j\in J}e\left(j,s_{1}\right)\cdot e\left(j,s_{2}\right)}{max(\sum_{j\in J}e\left(j,s_{1}\right),\sum_{j\in J}e\left(j,s_{2}\right))} \end{array}$$ (2)

where *e*(*j*, *s*) is the effective use of a skill in a job, defined as *e*(*j*, *s*) = 1 when *RCA*(*j*, *s*)≥1 and *e*(*j*, *s*) = 0 otherwise. Note that *θ*(*s*_1_, *s*_2_)∈[0, 1], a larger value indicates that *s*_1_ and *s*_2_ are more similar, and it reaches the maximum value when *s*_1_ and *s*_2_ always co-occur (i.e. they never appear separately) while *e*(*j*, *s*_1_) = 1 and $e(j,s_{2})=1,\forall j\in J$. Visibly, *θ*(*s*_1_, *s*_2_) is based on the co-occurrence of skills when both *s*_1_ and *s*_2_ are simultaneously important for the job ads. Therefore, *θ* measures when two skills are effectively used together—i.e., it measures similarity as in “complementary”, not as in “replaceable”.

### Skills Space method

Next, we use the pairwise skill distances to measure the distance between *sets of skills*, which we refer to as Skills Space. Here, a set of skills can be arbitrarily defined, such as an occupation, an industry, or a personalized skills set. Intuitively, two sets of skills are similar when their most important skills are similar. We first introduce a measure of skill importance within a skill set as the mean RCA over all the job ads pertaining to the skill set. Assume a job ads grouping criterion exists, for example, job ads pertaining to an occupation, a company, or an industry. We obtain the job ads set J⊂J and the set of skills S⊂S occurring within j∈J. We denote J as the set of job ads associated with the skill set S. We measure the importance of skill *s* for S (and implicitly for J) as the mean RCA over all the job ads relating to the skill set S. Formally,

$$\begin{array}{c} w\left(s,S\right)=\frac{1}{\left|J\right|}\sum_{j\in J}RCA\left(j,s\right) \end{array}$$ (3)

Next, we propose a method to measure the distance between *sets of skills*. For example, suppose there are two jobs that we can define by their underlying skill demands. Both jobs have their unique set of skills, and each individual skill has its own relative importance to the specific job, as calculated by Eq (3). Intuitively, the two jobs are similar when their most important skills (i.e., their ‘core’ skills) are similar. This is achieved by computing the weighted pairwise skill similarity between the individual skills of each job (using Eq (2)), where the weights correspond to the skill importance (defined by Eq (3)). This returns a single similarity score between the two skill sets corresponding to the two jobs. Formally, let $S_{1}$ and $S_{2}$ be two sets of skills, and $J_{1}$ and $J_{2}$ their corresponding sets of job ads. We define Θ the similarity between $S_{1}$ and $S_{2}$ as the weighted average similarity between the individual skills in each set, where the weights correspond to the skill importance in their respective sets. Formally,

$$\begin{array}{c} \Theta \left(S_{1},S_{2}\right)=\frac{1}{C}\sum_{s_{1}\in S_{1}}\sum_{s_{2}\in S_{2}}w\left(s_{1},S_{1}\right)w\left(s_{2},S_{2}\right)\theta \left(s_{1},s_{2}\right) \end{array}$$ (4)

where $C=\sum_{s_{1}\in S_{1}}\sum_{s_{2}\in S_{2}}w\left(s_{1},S_{1}\right)w\left(s_{2},S_{2}\right)$. Similar to *θ* defined in Eq (2), Θ is a similarity measure (higher means more similar) and $\Theta \left(S_{1},S_{2}\right)\in \left[0,1\right]$. Note that Θ is a compound measure based on *θ*, which in turn measures the complementarity of two skills (see prior discussion and interpretation of *θ*). As a result, $\Theta (S_{1},S_{2})$ measures the complementarity of two skill sets. The interpretation we use in the rest of this paper is that “when Θ is high, an individual with $S_{1}$ can more readily fulfil the skill requirements of $S_{2}$”. We use Θ as a key feature in our job transitions recommender system. The setup and details of this system now follow.

### Job transitions recommender system setup

We construct the job transitions recommender system as a binary machine learning classifier using XGBoost—an implementation of gradient boosted tree algorithms, which has achieved state-of-the-art results on many standard classification benchmarks with medium-sized datasets [27]. The XGBoost algorithm is a linear combination of decision trees where each subsequent tree attempts to reduce the errors from its predecessor. This allows for the next tree in the series ‘learn’ from the errors of the previous tree with the goal of making more accurate predictions. In our application of the XGBoost algorithm, the system ‘learns’ from the input labor market data, which are independent variables (or features). It is then ‘trained’ against historic examples of occupational transitions that did occur (positive examples) and did not occur (negative examples), which are the dependent variables (or ground-truth). As is standard in machine learning practice, we reserve a ‘test set’ of observations for evaluation, where we apply the trained model to make predictions about whether a transition occurred or not (hence, binary) by only observing the features. This setup allows us to predict the probability of an occupational transition from the ‘source’ to the ‘target’ occurring (positive example) or not (negative). Here, we use the job-to-job transitions data from the HILDA dataset [14] (described above). We then randomly simulate an alternate sample of transitions where we maintain the same ‘source’ occupations and randomly select ‘target’ occupations (called ‘Random Sample’). This produces a balanced dataset of 5,998 positive and negative occupational transitions. We then associate each ‘source’ and ‘target’ observation with their temporal pairwise distance measure using the Skills Space method. However, the Skills Space measures are symmetric, and job transitions are known to be asymmetric [9, 13, 28]. Therefore, to represent the asymmetries between job transitions, we add a range of explanatory features to each ‘source’ and ‘target’ occupation. These occupational features include their Skills Space pairwise distance measures and other variables, such as years of education required, years of experience demanded, and salary levels, from employment statistics (‘Labor Supply’) and job ads data (‘Labor Demand’—see S1 File for full list of features).

Like most machine learning algorithms, XGBoost has a set of hyper-parameters—parameters related to the internal design of the algorithm that cannot be fit from the training data. The hyper-parameters are usually tuned through search and cross-validation. In this work, we employ a Randomized-Search [29] which randomly selects a (small) number of hyper-parameter configurations and performs evaluation on the training set via cross-validation. We tune the hyper-parameters for each learning fold using 2500 random combinations, evaluated using a 5 cross-validation. We train the models on 80% of the observations, leaving aside 20% of the data for testing, which we randomly seed. We repeat the process 10 times for each feature model configuration and change the random seed to select a new testing sample, which provides us with the standard deviation bars seen in Fig 3.

### Constructing a leading indicator of AI adoption

Adapting the Skills Space method, we develop a leading indicator for emerging technology adoption and potential labor market disruptions based on skills data, using AI as an example. We select AI because of its potential impacts on transforming labor tasks and accelerating job transitions [2, 4, 6]. Our indicator temporally measures the similarity between a dynamic set of a AI skills against the 19 Australian industry skill sets from 2013–2019.

To create these yearly sets of top AI skills, we first select a sample of core ‘seed skills’ that are highly likely to remain important to AI over time—here we selected ‘Artificial Intelligence’, ‘Machine Learning’, ‘Data Science’, ‘Data Mining’, and ‘Big Data’ as the seed skills. This set of seed skills represents S from Eq (3) as opposed to a grouping criterion, such as an occupation or industry. In this case, J is not defined, and we measure the importance of a skill as the average *θ* similarity to the seed skills. Repeating this process temporally allows us to build dynamic skill sets. We then use Eq (2) to measure the similarity (*θ*) of each seed skill to every other skill in a given year. By calculating the average *θ* for every skill relative to the seed skills, we return an ordered list of skills with the highest similarity. This process is repeated for each calendar year from 2013–2019 where we select the top 100 skills for each year. The skill similarity approach allows us to build an adaptive list of AI skills that captures evolving skill demands. This is especially important for a skill area like AI, where the skill demands are changing very quickly. For example, ‘TensorFlow’ (a Deep Learning framework) emerged as a skill in November 2015 and has since become among the fastest-growing AI skills. The AI skill lists we create can detect the importance of ‘TensorFlow’ in 2016, whereas a static list pre-defined before 2016 would have missed these important changes to AI skill demands.

Having constructed temporal sets of AI skills, we then measure the yearly similarities between the AI skillsets and the skill sets of Australia’s 19 major industries—classified according to the Australian & New Zealand Standard Industrial Classification (ANZSIC) Division level. Using the Skills Space method, we construct each industry as a set of skills for every year and use Eq (4) to calculate similarity to the yearly AI skill sets. This allows us to observe and compare the extent to which AI skills have diffused throughout industries and the relative importance of AI skills to these industries. The advantages of using this skill similarity approach as opposed to *ad hoc* skill counts from pre-defined skills are twofold. First, we create dynamic sets of skills that capture evolving skill demands. Second, we account for skill importance within individual job ads by normalizing for high-occurring skills (see S1 File for more details).

## Results & discussion

### Skill similarity results

**Fig 1:**
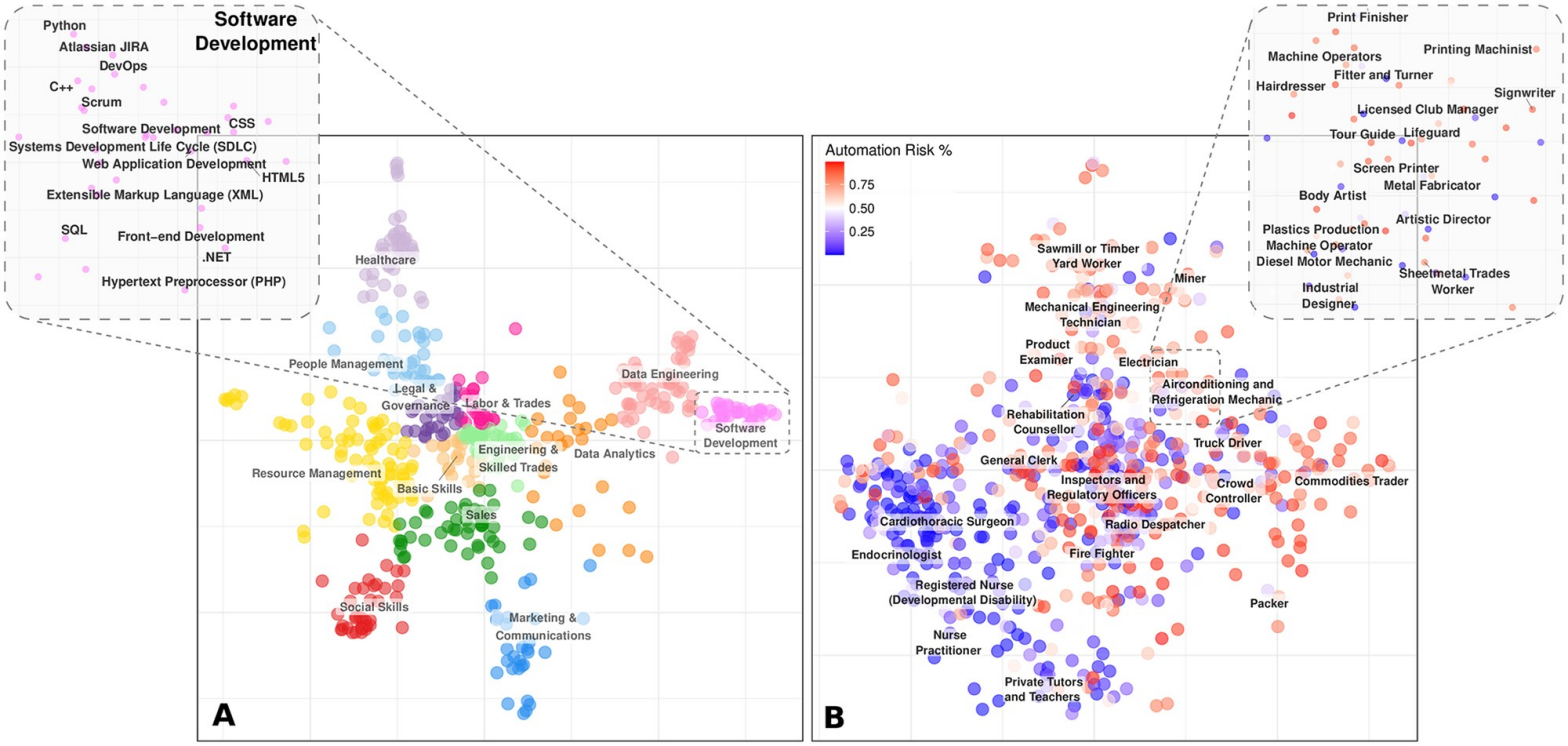
Measuring the distance between skills and occupations. (A) Shows a two-dimensional embedding of the 2018 skill distances, where the top 500 skills by posting frequency are visualized. Each marker represents an individual skill colored according to 13 clusters using K-Medoids clustering. As observed in the ‘Software Development’ inset, highly similar skills cluster together. Additionally, the specialized skill clusters, such as ‘Software Development’ and ‘Healthcare’ tend to lay toward the edges; whereas the more general and transferable skills lay toward the middle of the embedding and act as a ‘bridge’ to specialist skills. These individual skill distances form the basis of measuring the distance between sets of skills. (B) We leverage Skills Space to measure the distance between official Australian occupations at the 6-digit level (characterized by their skill sets) in 2018. The markers represent individual occupations, colored by technological labor automation risk, as calculated by Frey & Osborne. Occupations that require higher levels of routine, manual and/or low cognitive labor tasks tend to be at higher risk (colored darker red); whereas occupations characterised by non-routine, interpersonal, and/or high cognitive labor tasks are at lower risk (colored darker blue) over the next two decades.

Fig 1A shows the two-dimensional skill distance embeddings for the top 500 skills by posting frequency in 2018. Here, each marker represents an individual skill that is colored according to one of 13 clusters of highly similar skills, using the K-Medoids clustering algorithm. By using a triplets method for dimensionality reduction [30], we are able to preserve the global structure of the embedding (global structure = 98%). That is, two markers are depicted closer together when their corresponding skills are more similar (i.e., have low distance). This provides useful insights, highlighting that specialized skills (such as ‘Software Development’ and ‘Healthcare’) tend to lay toward the edges of the embedding, whereas more general and transferable skills lay toward the middle, acting as a ‘bridge’ to specialist skills. Highly similar skills cluster closely together; for example, the ‘Software Development’ cluster (see inset) regroups programming skills such as scripting languages ‘Python’, ‘C++’, and ‘HTML5’. It is important to measure the similarity between jobs based on their underlying skills because workers leverage their existing skills to make career changes [31].

### Skills Space results

In Fig 1B, the markers depict groups of skills that correspond to individual occupations, using the official Australian standard (at the 6 digit level—see S1 File for more details). To visualize the distance between occupations, we use the same dimensionality reduction technique as for individual skills in Fig 1A. Each occupation is colored on a scale according to their automation susceptibility, as calculated by Frey and Osborne [4]—dark blue represents low-risk probability, and dark red shows high-risk probability over the coming two decades. As seen in Fig 1B and the magnified inset, similar occupations lie close together on the map. Furthermore, occupations at low risk of automation tend to be characterized by non-routine, interpersonal, and/or high cognitive labor tasks [32]. In contrast, occupations at high risk of automation require routine, manual, and/or low cognitive labor tasks. For example, in the inset of Fig 1B, a ‘Sheetmetal Trades Worker’ is deemed to be at high risk of labor automation (82% probability) due to high levels of routine and manual labor tasks required by the occupation. However, a ‘Sheetmetal Trades Worker’ skillset demands are highly similar to an ‘Industrial Designer’, which is considered at low risk of labor automation over the coming two decades (4% probability). Therefore, an ‘Industrial Designer’ represents a transition opportunity for a ‘Sheetmetal Trades Worker’ that leverages existing skills and protects against potential risks of technological labor automation.

### Validation of Skills Space distance

**Fig 2:**
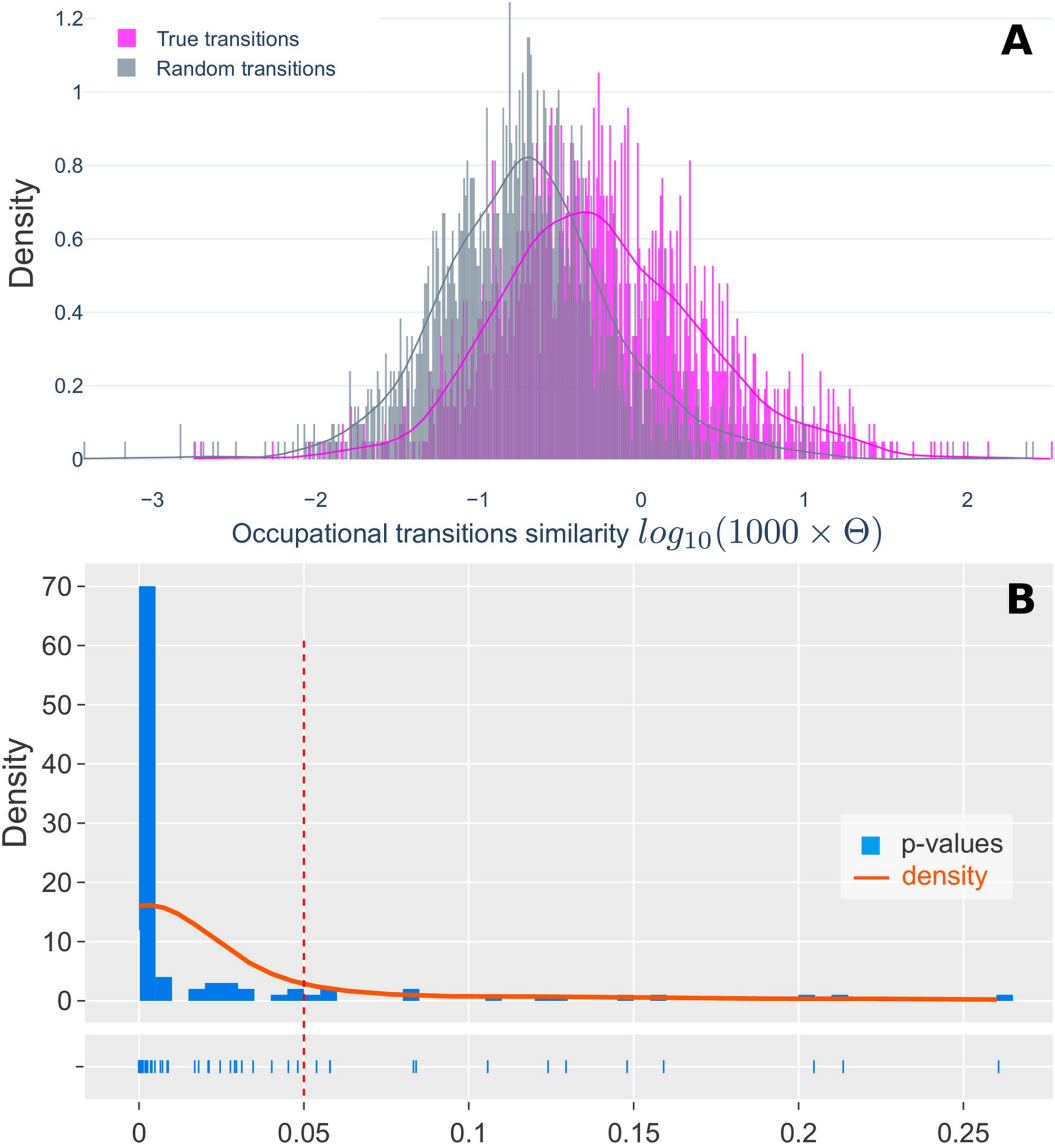
The Skills Space is statistically significant in representing job transitions. **(A)** The x-axis shows the log-transformed Skills Space distance for a ‘True’ sample of actual transitions (shown in magenta color) and a ‘Random’ sample of simulated transitions (shown in gray color). Each Random transition is paired with an Actual transition: it shares the same ‘source’ occupation as the Actual transition but the ‘target’ occupation is randomly selected and is different to the Actual observation. The difference between the two populations is statistically significant (paired t-test, t-statistic = 4.514, p-value = 6.535 × 10^−6^, Cohen’s D effect size = 0.14). **(B)** We repeat the procedure 100 times: we generate 100 ‘Random’ populations, and we perform the statistical testing for each. The figure shows the histogram (density and rug plot) of the 100 obtained p-values, 87 of which are lower than 0.05.

We validate the link between the Skills Space and job transitions by conducting paired t-tests, as illustrated in Fig 2. Here, we use a longitudinal household survey dataset containing actual job transitions [14] (called the ‘True Sample’). Each occupational pair (‘source’ to ‘target’) is labeled with its Skills Space distance for the given year. We randomly simulate an alternate transition sample by maintaining the same ‘source’ occupations and randomly selecting ‘target’ occupations (called ‘Random Sample’). Our objective is to determine whether the differences in Skills Space distance between the ‘True Sample’ and the ‘Random Sample’ are statistically significant. First, we test the differences of the two samples, including *all* occupational transitions. We find that the differences between the two samples are statistically significant (t-statistic = 16.272, p-value = 2.707 × 10^−58^, Cohen’s D effect size = 0.42) (see S1 File). However, one-third of the ‘True Sample’ transitions are to another job but are classified as the same occupation. Therefore, we perform a second test only on transitions between different occupations, i.e., we remove all observations where the ‘source’ and ‘target’ are identical. Again, the differences between the ‘True Sample’ and the ‘Random Sample’ are statistically significant (t-statistic = 4.514, p-value = 6.535 × 10^−6^, Cohen’s D effect size = 0.14), as illustrated in Fig 2A. We repeat the procedure 100 times: we generate 100 new ‘Random Samples’, and we perform the statistical test for each of them. 87% of these tests are statistically significant, as shown in Fig 2B. These results provide evidence that the Skills Space distance measure is representative of actual job transitions.

### Job transitions recommender system

**Fig 3:**
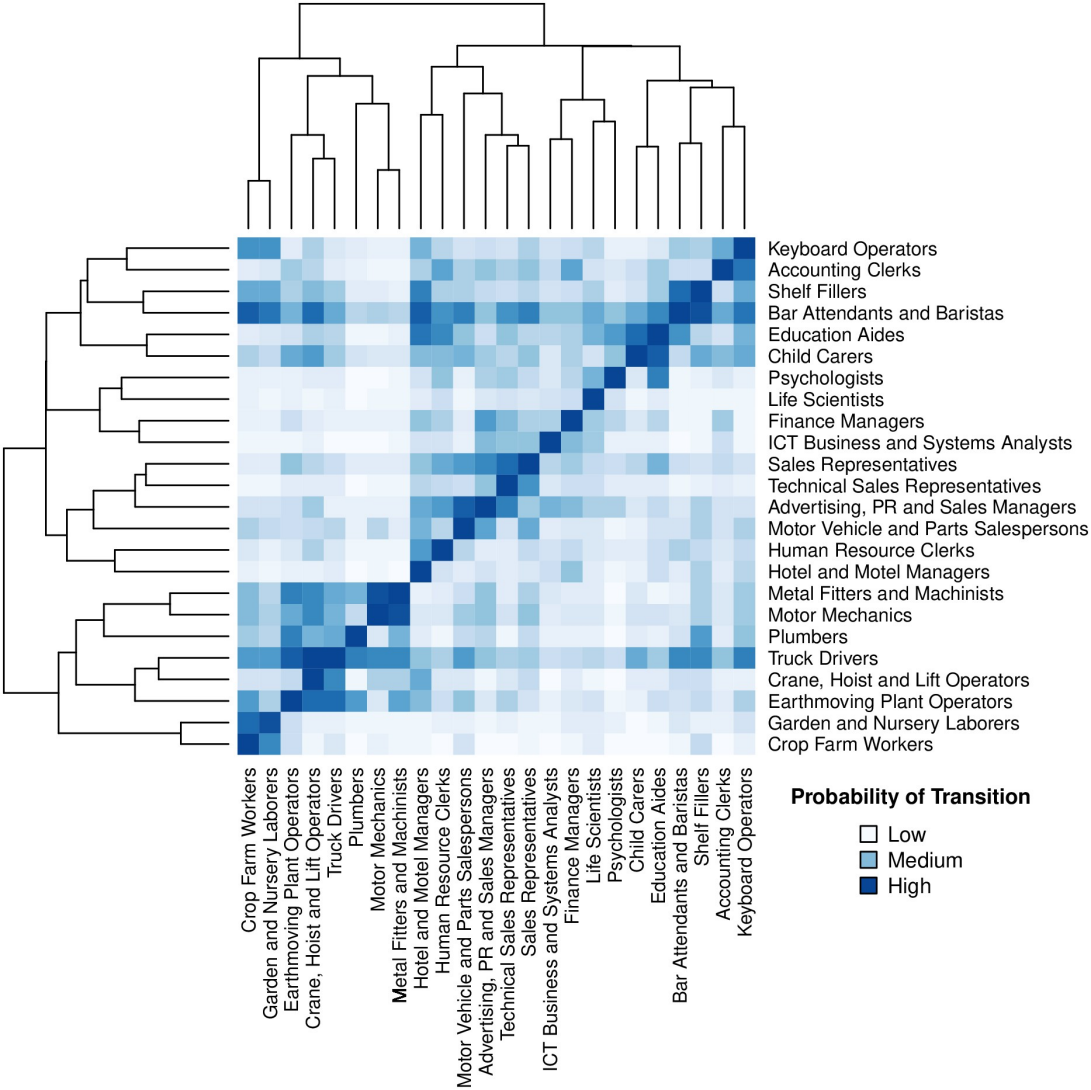
Validation and the *Transitions Map*. Visualizes a subset of the *Transitions Map*, where 20 occupations and their pairwise transition probabilities can be observed. In this visualization, transitions occur from columns to rows, and dark blue depicts high transition probabilities, and white depicts low probabilities. While job transitions to the same occupation yield the highest probabilities (dark blue diagonal squares), it is clear that transitions are asymmetric. The dendrogram highlights how similar occupations cluster together, where there is a clear divide between services and manual labor occupations.

Job transitions, however, are *asymmetric* [9, 13, 28]—the direction of the transition affects the difficulty. Therefore, transitions are determined by more than the symmetric distance between skill sets; other factors, such as educational requirements and experience demands, contribute to these asymmetries. We account for the asymmetries between job transitions by constructing a machine learning classifier framework that combines the Skills Space distance measures with other labor market features from job ads data and employment statistics (discussed in *Job Transitions Recommender System setup*). We then apply the trained model to predict the probability for every possible occupational transition in the dataset—described as the transition probability between a ‘source’ and a ‘target’ occupation. This creates the *Transitions Map*, for which a subset of 20 occupations can be seen in Fig 3. The colored heatmap shows the transition probabilities (‘source’ occupations are in columns and ‘targets’ are in rows). Dark blue represents higher transition probabilities, and lighter blue shows lower probabilities, where the asymmetries between occupation pairs are clearly observed. For example, a ‘Finance Manager’ has a higher probability of transitioning to become an ‘Accounting Clerk’ than the reverse direction. Moreover, transitioning to some occupations is generally easier (for example, ‘Bar Attendants and Baristas’) than others (‘Life Scientists’). The dendrogram illustrates the hierarchical clusters of occupations where there is a clear divide in Fig 3 between service-oriented professions and manual labor occupations.

**Fig 4:**
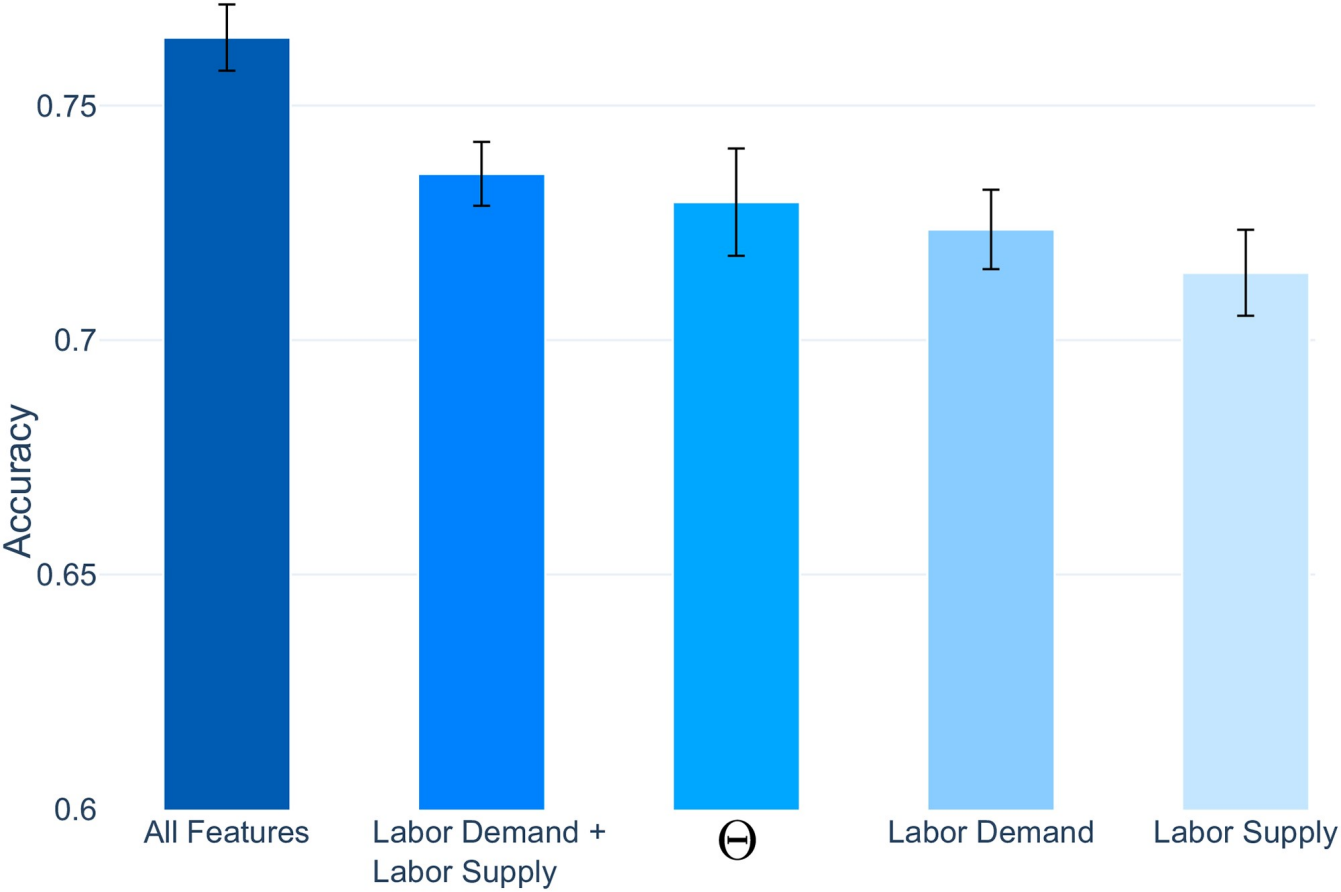
The prediction accuracy scores of the different classifier model feature configurations. The highest performance is achieved when ‘All Features’ are incorporated in the classifier models to predict occupational transitions (76%). Moreover, by incorporating all features, the standard deviation decreases (shown by the performance bars), which highlights the complementarity of the combined features and the ability to now account for the asymmetry between jobs.

For validation, we train various classifier models with different feature configurations and identify three main findings. *First*, as seen in Fig 4, the models that incorporate the distance measures with all of the other occupational features (‘All Features’) consistently achieve the highest accuracy for occupational transitions (Accuracy = 76%). This feature setup achieves higher results than models that only use the ‘Labor Demand + Labor Supply’ features (Accuracy = 74%) or the distance measures alone (Accuracy = 73%). To further validate these findings, we conduct an ablation test where each feature is iteratively removed from the feature set and the model is re-trained—the model configurations with lower performance indicate higher feature importance. Here, the exclusion of the Skills Space distance measure caused the greatest decline in performance, therefore reiterating its predictive power. We also conduct a feature importance analysis, which again shows that the Skills Space distance measure is the most important feature for predicting transitions (see S1 File for further details). *Second*, the standard deviation of accuracy over repeated trials decreases when all features are combined (as seen with the spread of the performance bars in Fig 4). This shows that the Skills Space distance measures and the occupational features are complementary in predicting job transitions. *Third*, and most important, is that by combining all features, we can construct the asymmetries between occupations. While transitioning to a job in the same occupation yields the highest probabilities (as seen by the dark blue diagonal line in Fig 3), the occupational features add asymmetries between occupational pairs, such as accounting for asymmetries in education and experience requirements.

#### Recommending jobs and skills

**Fig 5:**
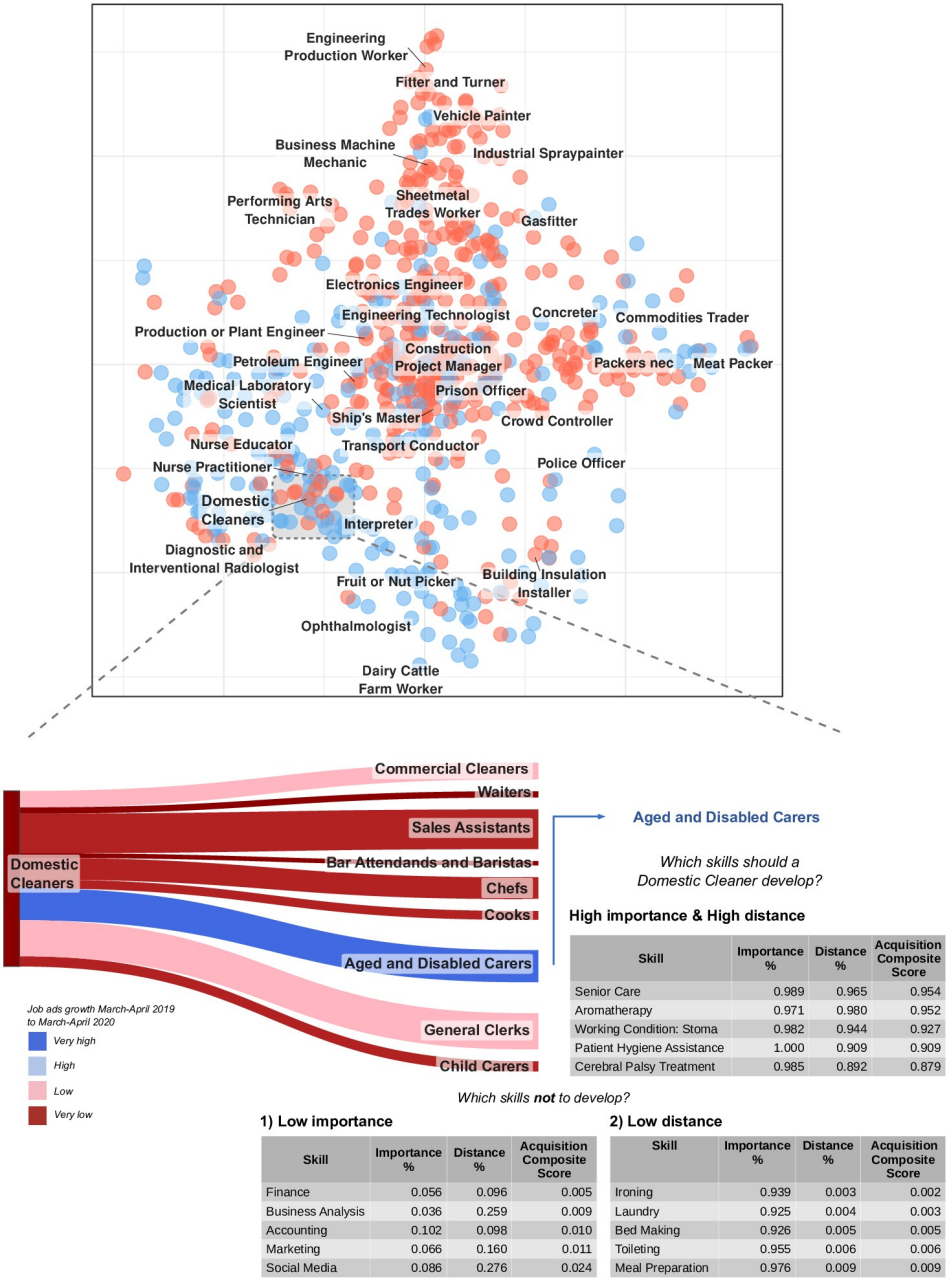
Here, we demonstrate the *Job Transitions Recommender System* using the ‘Domestic Cleaner’ occupation as an example—An occupation classified as a ‘non-essential’ occupation and that has experienced significant declines during the beginning of the COVID-19 outbreak in Australia. (upper panel) The two-dimensional space of occupations (see Fig 1) with ‘essential’ occupations in blue markers and ‘non-essential’ occupations in red markers. (lower panel) We first use the *Transitions Map* to calculate the occupations with the highest transition probabilities (other than itself). These are the nodes on the right side of the flow diagram in the bottom panel of the figure, where the link colors depict posting frequency percentage change from March-April 2019 to March-April 2020. The link widths represent the posting frequency volume of March-April 2020 to indicate labor demand. The top six occupations have all experienced significant declines during the COVID-19 period; however, the seventh recommendation, ‘Aged and Disabled Carers’, is experiencing significant labor demand growth. ‘Aged and Disabled Carers’ were also classified as an ‘essential’ occupation during COVID-19 in Australia. We select this as the target occupation and then make personalized skill recommendations. We argue that workers trying to transition to another occupation should invest time and resources into skill development when (1) the skills are of **high importance** *and* (2) there is a **high distance** to acquire the skill. Conversely, workers should *not* focus on skill development if (1) the skills are **low importance** *or* (2) there is a **low distance** to acquire the skill.

The *Transitions Map* provides the basis for making qualified job transition recommendations. We call this the *Job Transitions Recommender System*. In Fig 5, we showcase its usage in the context of a labor market crisis (i.e. COVID-19). We highlight the example of ‘Domestic Cleaners’, an occupation that experienced significant declines in labor demand and employment levels during the crisis (see S1 File).

During the ‘first wave’ of COVID-19 in March 2020, the Australian Government enforced social-contact and mobility restrictions on ‘non-essential services’ to slow the spread of the virus [33]. Many occupations within these ‘non-essential’ services were unable to trade and perform their duties, forcing some workers to try and transition to another job. In the upper panel of Fig 5, we visualize the ‘essential’ and ‘non-essential’ occupations on the *Transitions Map*. The placement of the occupations is identical to Fig 1B and we highlight the ‘essential’ occupations as the blue nodes and the ‘non-essential’ occupations are the red nodes using the classifications developed by Faethm AI [34]. We observe that the cluster of medical occupations (bottom-left of the map) are deemed as ‘essential’, as are most of the food production workers (bottom). Here, we select ‘Domestic Cleaners’ as an example of a ‘non-essential’ occupation and use the *Transitions Map* to recommend the occupations with the highest transition probabilities in the bottom panel of Fig 5. These are the nodes on the right side of the flow diagram in Fig 5, ordered in descending order of transition probability. Segment widths show the labor demand for each of the recommended occupations during the COVID-19 period (measured by posting frequency). The segment colors represent the percentage change of posting frequency during March and April 2019 compared to the same months in 2020; dark red indicates a big decrease in job ad posts, and dark blue indicates a big increase. The first six transition recommendations for ‘Domestic Cleaners’ have all experienced negative demand, which is unsurprising given that they were also deemed as ‘non-essential’ services. However, the seventh recommendation, ‘Aged and Disabled Carers’, has significantly grown in demand during the COVID-19 period, and there is a high number of jobs advertised. ‘Aged and Disabled Carers’ is both an ‘essential’ and a high-demand occupation; therefore, it makes sense to select ‘Domestic Cleaner’ as the target occupation for transitioning into.

We take the *Job Transitions Recommender System* a step further by incorporating skill recommendations. Transitioning to a new occupation generally requires developing new skills under time and resource constraints. Therefore, workers must prioritize which skills to develop. We argue that a worker should invest in developing a skill when (1) the skill is important to the target occupation *and* (2) the distance to acquire the skill is large (that is, it is relatively difficult to acquire). Formally, for a target skill (i.e., a skill in the ‘target’ occupation), we compute its *importance* to the target occupation and its *distance* to the source occupation. We calculate *skill importance* as the mean *RCA* for the skill across all job ads within the target occupation using Eq (3). We calculate *skill distance* as the distance between the target skill and ‘source’ occupation skill set as 1 − Θ(*S*_1_, *S*_2_) using Eq (4) (i.e., the ‘target’ skillset (*S*_2_) is made out of a single skill: the target skill). Finally, we build the *acquisition composite score* as the multiplication of importance and distance, transformed as percentiles to account for variation in scale.

In the case of the ‘Domestic Cleaner’ in Fig 5 (lower panel), the skills with the highest acquisition composite score for the transition to ‘Aged and Disabled Carer’ are specialized patient care skills, such as ‘Patient Hygiene Assistance’. Conversely, the reasons not to develop a skill are when (1) the skill is not important *or* (2) the distance is small to the target occupation. Fig 5 (lower panel) shows that while some ‘Aged and Disabled Carer’ jobs require ‘Business Analysis’ and ‘Finance’ skills, these skills are of low importance for the ‘Aged and Disabled Carer’ occupation, so they should not be prioritized. Similarly, skills such as ‘Ironing’ and ‘Laundry’ are required by ‘Aged and Disabled Carer’ jobs, but the distance is small, so it is likely that either a ‘Domestic Cleaner’ already possesses these skills or they can easily acquire them. Visibly, for both of the latter cases, the acquisition composite score takes low values.

### A leading indicator of AI adoption

Emerging technologies can change the demand for labor and accelerate forced job transitions by disrupting labor markets [35]. However, in order for emerging technologies to have these effects, they must first be widely adopted by firms across many industries. In this sense, technology adoption rates are a precursor to the societal impacts that they impose, such as widespread changes to labor demand and accelerated job transitions. Measuring technology adoption, however, can be difficult as it often depends on the private activities of firms and is influenced by a range of factors (see S1 File). Therefore, measuring the drivers that enable emerging technology adoption (see S1 File) can provide leading indicators of adoption rates. One major driver is the availability of skilled labor. Firms that can readily access workers with relevant skills are able to make productive use of the emerging technologies and accelerate their adoption rates, particularly in the early stages of technology growth [36]. Skills Space offers a useful method for identifying the extent of specific skill gaps of firms within industries. As an industry’s skills set becomes more similar to the skills of an emerging technology, the skills gap is narrowed, and the barriers to adopting the emerging technology are reduced. When access to relevant skilled labor is plentiful, the labor requirements enabling technological adoption can be readily achieved and help accelerate adoption rates. Therefore, measuring temporal levels of skill set similarity for an emerging technology within an industry provides a useful leading indicator of technology adoption over time. These measures offer early detection signals of emerging technology adoption and the changing skill requirements that could cause labor disruptions within industries, such as forced job transitions.

**Fig 6:**
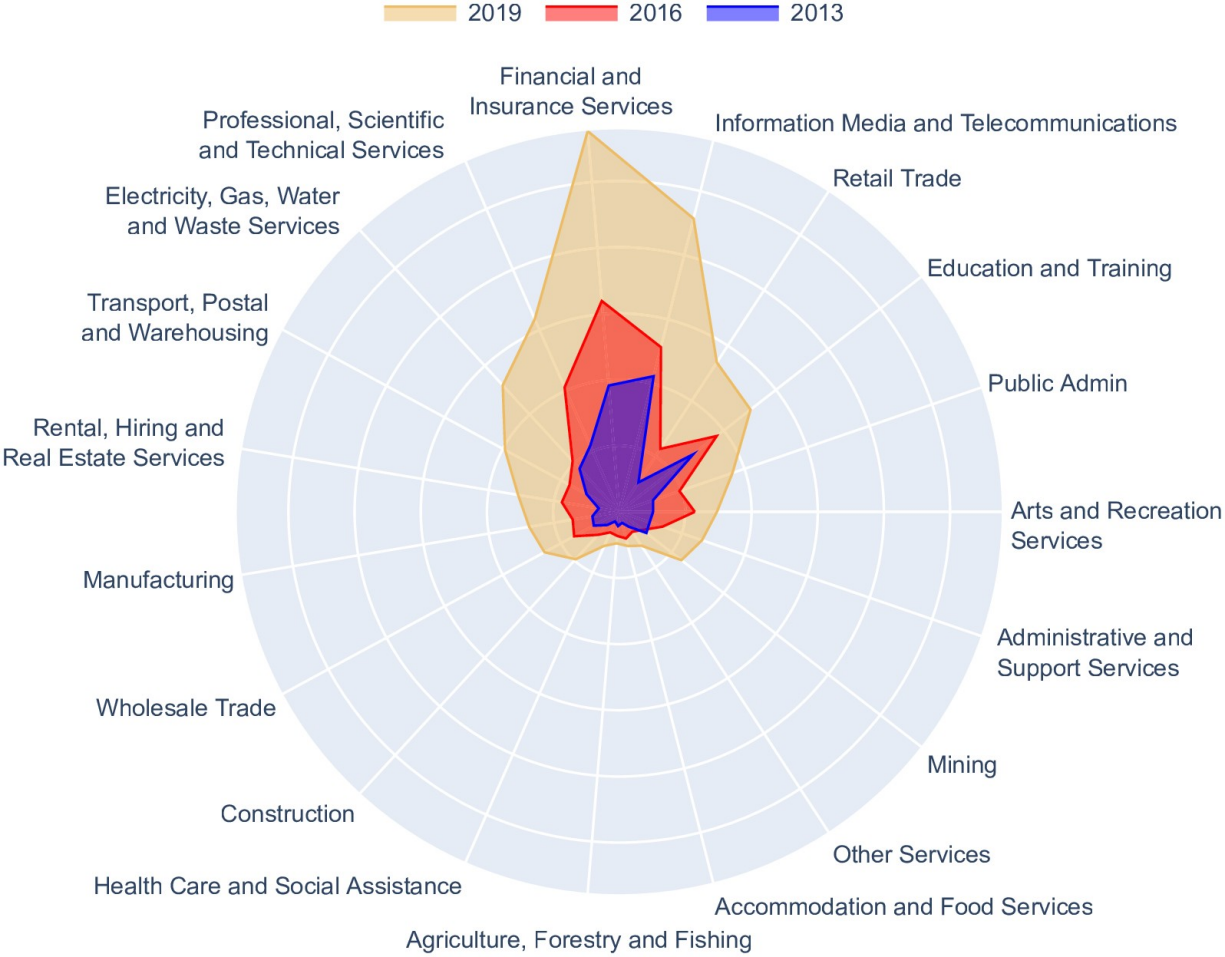
By applying Skills Space, we measure the yearly similarities between adaptive sets of AI skills against each of the 19 Australian industries from 2013–2019. As industry skill sets become more similar to AI skills, the colored area of the radar chart expands. All industries have increased their similarity levels to AI skills, albeit at different rates. We argue that higher levels of AI similarity indicate AI skills are becoming more important to firms within an industry and that the skills gap to acquiring AI skills is narrowed. Access to these skills accelerates the rate of firms adopting AI and making productive use of the technologies, which offers a leading indicator of AI adoption and potential labor disruptions within these industries.

Fig 6 shows that all Australian industry skill sets have grown in similarity to AI skills from 2013 to 2019—illustrated by the expanding colored areas. This highlights the growing importance of AI skills across the Australian labor market. However, the rates of similarity are unequally distributed. Some industries—such as ‘Finance and Insurance Services’ and ‘Information Media and Telecommunications’—command much higher rates of AI skill similarity. This indicates that not only are firms within these industries increasingly demanding AI skills but also that the AI skills gaps within these industries are much smaller. Also noteworthy are the differences in growth rates toward AI skill similarity. As clearly seen in Fig 6, AI skill similarity has rapidly grown for some industries and more modestly for others. For instance, ‘Retail Trade’ has experienced the highest levels of growth in similarity to AI skills, increasing by 407% from 2013 to 2019. The majority of this growth has occurred recently, which coincides with the launch of Amazon Australia in 2017 [37]. Since then, Amazon has swiftly hired thousands in Australia.

By adapting the Skills Space method, we develop a leading indicator that detects AI adoption from real-time job ads data. Such a measure can act as an ‘early warning’ signal of forthcoming labor market disruptions and accelerated job transitions caused by the growth of AI. This indicator can assist policy-makers and businesses to robustly monitor the growth of AI skills (or other emerging technologies), which acts as a proxy for AI adoption within industries (or other labor market groups).

### Limitations

We acknowledge several limitations of the Skills Space method and the results presented in this paper. First, there are *data limitations* from both the household survey data and the job ads data. The job transitions drawn from the HILDA panel dataset are a relatively small sample, with 2,999 job transitions from 2012–2018. While these observations come from the high-quality HILDA dataset, which is Australia’s pre-eminent and representative household survey [14], it is nonetheless a relatively small sample to train a machine learning system against. A small sample enlarges the risks of biases emerging as the recommender system is dependent on a relatively small sample of observations to ‘learn’ from and make predictions about future job transitions. Longitudinal household surveys also suffer from panel attrition, including HILDA [38]. However, this is only a minor issue for this study, as yearly job transitions are treated as independent observations. Our methods are not dependent on the longitudinal career pathways of individuals. With regards to the job ads data, we only had access to the Australian job ads dataset. As a result, our analyses and results are specific to the Australian labor market. However, this is a feature of our work rather than a limitation, as it allows to contextualize the analysis to geographical and temporal labor markets—it has been shown that labor markets can be highly contextual [39]. One can easily leverage our methods to produce results for other countries by applying equivalent country-specific labor market data from job ads, employment statistics, and occupational transitions.

Second, the results presented in this paper have been *aggregated to the occupational level*. That is, the explanatory features have been grouped by their 4-digit occupations, such as median salary and average education for a given occupation (see the S1 File for a full list of the features). Consequently, the job transition predictions in this paper are made at the grouped 4-digit occupational level for demonstration purposes, which does not differentiate within the same occupations. However, the flexibility of the methods presented in this paper can be applied at the individual level (or another arbitrary grouping), given the availability of appropriate data sources.

Third, there can be many factors that cause individuals to transition between jobs [13], beyond those used as explanatory variables in this study. For example, it has been shown that personality profiles are predictive of different occupational classes [40]. Therefore, it is plausible that personality traits and values could influence not only the willingness to move between jobs but also the types of job transitions. Similarly, there exists a Markovian assumption that a worker is described by the set of skills in their current job, therefore ignoring their past work experience and education. Other factors such as competitive dynamics within specific industries and labor markets, macroeconomic conditions, and changes to individuals’ household finances can all influence people transitioning between jobs. Future work could look to incorporate these additional variables to help further explain and predict job transitions.

Last, we must acknowledge the risks of biases emerging from applying mechanical algorithms to ‘learn’ from historical examples and make consequential recommendations to people, such as suggested career pathways. If the data used to train a machine learning system contains biases, then the predictions generated by the system are likely to reflect these biases. For example, there are structural biases in labor markets that influence employment outcomes, including biases based on gender [41], race [42], age [43] and others. As the Australian labor market is not immune to systemic biases [44], likely, the training data used for this research (HILDA—a representative household survey) reflects these systemic biases to some extent. Therefore, the results presented in this paper should be viewed as ‘descriptive’ of labor mobility in Australia rather than ‘prescriptive’ of individuals’ career options. To help safeguard these systemic biases, we add a human-in-the-loop to filter the generated recommendations. The system we design in Fig 5 is a decision-aid tool that filters top recommendations based on posting frequency (the link colors) to identify which occupations are growing in demand. Additional filters can be applied, such as top recommendations based on salary, education level, years of experience demanded, specific skill sets, industries, and others. While these filters do not remove biases from the recommendations entirely, they do provide individuals using this system with greater autonomy in exploring potential career paths.

## Conclusion

Leveraging longitudinal datasets of real-time job ads and occupational transitions from a household survey, we have developed the Skills Space method to measure the distance between sets of skills. This enabled us to build systems that both *recommend* job transition pathways based on personalized skill sets and *detect* the growth of disruptive technologies in labor markets that could accelerate forced job transitions. Our *Job Transitions Recommender System* has the potential to assist workers, businesses and policy-makers to identify efficient transition pathways between occupations. These targeted and adaptive recommendations are particularly important during economic crises when labor displacement increases and workers are forced to transition to another job. The *Job Transitions Recommender System* could therefore assist with the current labor crisis caused by COVID-19. Additionally, it could assist with potential future crises, such as accelerated job transitions caused by AI labor automation.

We further demonstrate the usefulness and flexibility of Skills Space by applying it as a measure of AI adoption in labor markets. This acts as an ‘early warning system’ of forthcoming labor disruptions caused by the adoption and diffusion of AI within industries. Such a measure could complement other indicators of AI adoption, serving policy-makers and businesses to monitor the growth of AI technologies and its potential to accelerate job transitions.

While the future of work remains unclear, change is inevitable. New technologies, economic crises, and other factors will continue to shift labor demands causing workers to move between jobs. If labor transitions occur efficiently, significant productivity and equity benefits arise at all levels of the labor market [45]; if transitions are slow, or fail, significant costs are borne to both the State and the individual. Therefore, it is in the interests of workers, firms, and governments that labor transitions are efficient and effective. The methods and systems we put forward here could significantly improve the achievement of these goals.

## Supporting information

S1 File (PDF)

S1 Fig Density plots for the statistical tests against all occupational transitions (A) and against transitions where the worker changed occupations (B). (TIFF)

S2 Fig Prediction performance and confusion matrix. Job transition model that includes all features achieves the highest results, as seen with (A) the confusion matrix and (B) the ROC curve; whereas the job transitions classifier model that only includes Skills Space distance method has lower performance, as seen by (C) the confusion matrix and (D) the ROC curve.
(TIFF)

S3 Fig Quantify feature importance. (A) Ablation test of classifier features and (B) feature importance analysis both show that the Skills Space distance measure (‘theta’) is the most important feature for predicting occupational transitions.
(TIFF)

S4 Fig Yearly skill similarities between AI skills and Australian industry (ANZSIC Division) skill sets from 2012–2019. (TIFF)

S5 Fig Empirical cumulative distribution function of skill counts within job ads for 2018. (TIFF)

S6 Fig Posting frequency of AI skills. Yearly posting frequency of the five AI seed skills used to build a dynamic list of yearly AI skills.
(TIFF)

S7 Fig Vacancy rate of AI skills. The percentage of vacancies in Australia that contain these five AI seed skills.
(TIFF)
