# Supplementary material for: Skill-driven recommendations for job transition pathways
Source: PLoS One. 2021 Aug 4;16(8):e0254722. doi: 10.1371/journal.pone.0254722 (PMC8336878; doi:10.1371/journal.pone.0254722)
Supplement: S1 File — (PDF) [file pone.0254722.s001.pdf]

This document is accompanying the submission Skill-driven Recommendations for Job Transition Pathways by authors Nikolas Dawson, Mary-Anne Williams, and Marian-Andrei Rizoiu. The information in this document complements the submission, and it is presented here for completeness reasons. It is not required for understanding the main paper, nor for reproducing the results.

S1 Appendix: Related Work

This section discusses the related works that have directly informed the research in Skill-driven Recommendations for Job Transition Pathways. There is firstly a discussion of the factors affecting labor mobility, the causes and effects of skill mismatches, measuring human capital transferability, and accounting for the asymmetries between jobs. Then there is a brief literature review of the factors affecting the adoption of Artificial Intelligence (AI) technologies.

Job Transitions

The related literature on job transitions broadly falls into the categories of labor mobility and human capital within the discipline of labor economics. 'Labor mobility' refers to the allocation of workers to firms and their ability to move between jobs [1]. Labor mobility is an important determinant of healthy labor markets. Efficient labor movements enable firms to hire more productive workers, effectively match workers to jobs based on their preferences, and helps to protect markets against economic shocks and structural changes. 'Human capital' refers to the skills, knowledge, capabilities, and experiences possessed by an individual that influence their productive capacities and that can be exchanged for labor at a prevailing market wage [2–4].

The process of labor mobility is constantly evolving and influenced by a variety of factors. These include labor market policies that shape hiring practices, job separation

support programs, and relocation incentives [5–7]. It is also impacted by the extent of human capital in a labor market, which refers to the supply of skills, knowledge, and abilities of a labor force that firms can employ to produce goods and services [8]. According to Nedelkoska and Frank, skills should be considered part of human capital that is acquired through education, training, and work experiences [9]. While access to education and training undoubtedly affects the acquisition of skills, particularly 'general' skills [3], skills are also acquired through work experience. Typically, firm or industry-specific skills are not perfectly mobile across employers and can hinder labor mobility [10]. Therefore, the extent of human capital 'specificity' in labor markets is an important factor affecting labor mobility. It impacts how transferable skill sets are between jobs and reveals their underlying mismatch. The remainder of this section reviews the literature relating to the cause, effects, and measurement of skill mismatch and skill transferability. There are subtle, but important, differences between these terms. 'Skill mismatch' refers to the differences between the supply of and demand for skills in a labor market. Whereas 'skill transferability' is the capacity to leverage previously acquired skills to perform tasks across different jobs, either because the tasks are similar or the skills can be flexibly applied to different tasks [9].The following literature forms the theoretical basis that directly informs our novel approach to measuring the distance between skills and sets of skills.

Causes and effects of skill mismatches.

Skills provide the means for workers to complete tasks that are required by jobs. A distinction should also be made between skills, knowledge areas, and abilities. 'Skills' are the proficiencies developed through training and/or experience [11]; 'knowledge' is the theoretical and/or practical understanding of an area; and 'ability' is the competency to achieve a task [12], where a task is a unit of work required by a job. For simplicity, the term 'skill' will collectively represent these three definitions throughout this paper.

Skill mismatch occurs when the skill demands of a job differ from the supply of skills available in a labor market [9]. When labor demand outweighs the supply of specific skills, it is referred to as a 'skill shortage'; when supply exceeds labor demand, it is often called 'skill excess' or 'over-supply'. Skill mismatches are closely monitored by labor economists due to the cost burdens they impose. For workers, they lower wages

and employment opportunities; for firms, they restrict access to talent to implement specific tasks; for economies, they drain productivity [9].

The causes of skill mismatches can be both frictional and structural. There are search costs associated with a worker finding a job suitable to their skills, education, and experiences [13]. The frictions of matching workers to appropriate jobs can hamper efficient job transitions and exacerbate skill mismatches. Structural factors causing skill mismatches, however, are concerned with the supply and demand for skills.

Regarding the supply side, the literature has mainly focused on the role of public institutions to facilitate skill development through education and training. Freeman, among others, demonstrated that the oversupply of skills can depress wage premiums that are typically earned by highly educated workers [14]. Further, Goldin and Katz showed that the wage premium of US college graduates was a function of the supply of college education, with college wage premiums increasing when the supply of college degrees in the labor market was low [15].

Structural changes in the demand for skills are predominantly caused by (1) technological advances and (2) globalization or trade. Concerning technology and innovation, Vona and Consoli [16] present a useful framework for understanding the evolving relationship between skills and technological change. In the early stages of new technology adoption, the authors argue that tasks are typically complex and non-routine. Consequently, specialized and highly skilled labor is required to make productive use of these new technologies. As time progresses, however, knowledge becomes structured and codified, enabling tasks to be routinized and automated. Eventually, the marginal benefits of specialization diminish as the use of the technology becomes standardized and tasks are able to be performed by lower-skilled workers. Related, is the theory of **Skill-Biased Technological Change** (SBTC). The SBTC hypothesis posits that technologies disproportionately advantage highly-skilled labor over lower-skilled labor, as technologies tend to enhance the skills highly-skilled workers and automate lower-skilled workers [17–19]. The SBTC hypothesis was later modified to account for the relationship between computers and task-specific requirements of jobs. This Task-biased Technological Change (TBTC) framework [20, 21] classifies labor tasks along two main spectra; routine to non-routine tasks and cognitive to manual tasks. Computerization, according to TBTC, tends to assist non-routine tasks and automate

routine tasks. As a result, computers automate the labor tasks of middle-skilled workers (typically, routine-cognitive workers), which helps to account for recent dynamics such as declining real wages of middle-skilled workers and labor polarization [22]. However, the argument that the negative demand-side effects of computerization are limited to routine tasks is now coming under scrutiny. The rapid advances and diffusion of AI technologies cast doubt over this assumption. Brynjolfsson and McAfee [23] and Frey and Osborne [24] present compelling arguments that the automation capabilities of AI are extending to non-routine tasks, both in the cognitive and manual domains. Non-routine tasks that were previously considered out of reach by AI are quickly outperforming human levels in a range of non-routine tasks, such as Natural Language Processing (NLP) [25], Image Recognition [26], and unstructured learning tasks [27].

Globalization or trade also has important implications on the demand for skills, which can exacerbate skill mismatches. Offshoring enables firms to fulfill their required labor tasks without personal contact, which can be managed electronically without a loss in quality [28]. This shifts the demand for skills towards countries with lower labor costs. Autor et al. [29] found that approximately one quarter of the decline in US manufacturing employment can be attributed to increasing trade with China.

Taken collectively, changes in the supply of and demand for skills alter the extent of skill mismatches in a labor market. This equilibrium is dynamic and directly affects labor mobility. The following subsection reviews the literature for measuring skill mismatch and transferability, which directly informs this work.

Measuring skill transferability and mismatch.

This research is part of a small but growing area of labor economics that measures the 'distance' between skills, jobs, and other defined skill sets. Among the earliest work in this area was conducted by Shaw [30, 31] who defined measures of occupational distances via proxies of skill transferability across occupations. This was based under the assumption that occupations with high levels of skill transferability are strongly correlated with high probabilities of transitioning between these occupations. This is an assumption that we adapt, test, and prove in Skill-driven Recommendations for Job Transition Pathways.

More recent studies have made use of skill and task-level data, such as the US

Dictionary of Occupational Titles (DOT - a predecessor to O*NET) or the German Qualification and Career Survey (QCS). Poletaev and Robinson [32] use task-level data from DOT to study the similarity between occupations. The authors construct four measures of basic skills, applying the factor analysis method used by Ingram and Neumann [33]. These four skill measures characterize the 'skill portfolios' of occupations, which are organized as vectors of skills. They then use Euclidean distance to compute the similarity between occupational skill vectors in order to identify which workers change their skill portfolios when transitioning between jobs. The authors show that workers who find jobs with similar skill requirements to their earlier jobs before displacement avoid large wage losses.

Similarly, Gathmann and Schönberg [34] use the QCS to classify occupations into a 19-dimension skill space defined by the survey. Each occupation represents a skill vector, where occupations consist of certain skills with varying degrees of mastery. The authors use the angular distance between the 19 skill vectors to position the occupations and measure their relative distances. The authors demonstrate that individuals transition to occupations with similar task requirements and that the distance requirements decline with greater work experience.

Most recently, Alabdulkareem et al. [35] used techniques from Network Science and unsupervised Machine Learning to illustrate occupational polarization based on their underlying skill. Data sources included a combination of O*NET skill-level data and US occupational transitions data in the Current Population Survey from the US Bureau of Labor Statistics. The authors implemented an established measure from Trade Economics, called 'Revealed Comparative Advantage' (RCA), to firstly measure the relative importance of a skill in a job while normalizing for high-occurring skills. After setting a threshold for skill importance, skill similarity was then calculated as the minimum of conditional probabilities that a skill pair are both important in a job when they co-occur. The authors then used these pairwise skill similarities to map workplace skills as a network, highlighting skill polarization and proving a correlation with wage polarization. Dawson et al. [36] extended this approach by applying this method to real-time job ads data to adaptively select occupations based on their underlying skill demands. This enabled the authors to accurately monitor changing labor demands and detect skill shortages for an evolving set of Data Science and Analytics occupations in

Australia. The skill similarity methods applied by Alabdulkareem et al. [35] and Dawson et al. [36] provide the foundation for the Skills Space method.

While all of these approaches represent significant contributions in the evolution of measuring skill transferability, there is one major shortcoming. All of these methods yield symmetric distance measures. That is, the distance from one skill or occupation to another is the same despite the direction. For example, according to these methods, it is just as difficult for a Nurse to become a Surgeon as the other way around. Intuitively, however, acquiring certain skills to transition to a particular occupation is more difficult in one direction than the other. In this sense, skill acquisition and occupational transitions are directed and asymmetrical.

Asymmetric measures for skill mismatches.

Nedelkoska et al. [37] develop skill mismatch metrics that account for the strong asymmetries in the transferability between skills. The authors construct occupational skill profiles by using factor analysis to extract five task-based skills on German administrative and data on individuals' work histories. They then calculate the share of workers in each occupation carrying out these tasks. The average years of education and training associated with each task are used as weights to indicate skill complexity required by an occupation. Adding these weights reveals asymmetries between skills and therefore occupations. They show that by switching occupations, people incur both skill shortages and skill redundancies, which results in significant wages losses up to 15 years following the job displacement. While accounting for skill asymmetries represents a clear improvement for measuring the distance between skills, using years of education and training as the sole proxy for skill complexity is questionable. As previously stated, work experiences are an important contributor to the acquisition of skills and causes of mismatch.

Bechichi et al. [38] adapt the Nedelkoska et al. [37] model by analyzing occupational data from the OECD Survey of Adult Skills (PIAAC). They firstly use the six task-based skill indicators from PIACC [39]. The authors then measure these indicators on 127 occupations (at the 3-digit occupational level) across 31 different countries to assess occupational distances based on 'cognitive skills' and skills acquired from tasks 'on the job'. This method accounts for skill asymmetries and skills acquired from work

experiences. The resulting ‘skill shortage’ and ‘skill excess’ measures are then used to predict education and training resources required to transition workers from one occupation to another. Therefore, this research represents another advance toward the goal of accurately measuring skill and occupational distances. However, a minor shortcoming of this work is that it is performed at the 3-digit occupational level, which is a relatively high classification level (1-digit being the highest and 6-digit being the lowest and most detailed). Additionally, surveys provide lagging data that are typically slow to report and expensive to conduct. This is problematic in labor crises, such as the job displacements caused by COVID-19. Dynamics of labor markets quickly change in times of crisis and displaced workers are faced with transitioning between jobs with rapidly evolving skill demands. Real-time data, therefore, becomes essential.

Our research builds on these significant works and addresses both of these shortcomings by using real-time job ads data and applying a method capable of measuring the distance between any defined set of skills, such as occupations at the detailed 6-digit occupational level, industries, or even personalized skill sets.

S2 Appendix: Artificial Intelligence Adoption

The labor market impacts of AI depend on the adoption rates of AI technologies by firms. If firms are slow or fail to adopt AI, then its effects are naturally restricted. Therefore, the risks of AI accelerated labor automation will only be realized if these technologies are adopted by firms, absorbed in workflows, and broadly diffused. Otherwise, they’re just isolated use cases.

This consideration, however, is often ignored. Much of the recent research on the economic impacts of AI assume broad adoption and diffusion. For example, the prominent study by Frey and Osborne estimated that 47% of occupations face a near-term risk of automation from AI [24]. These results were based on the assessments of a small panel of Machine Learning experts who were asked to identify which of 70 jobs were ‘completely automatable’ in 2013. However, these forecasts rely on some questionable assumptions. Chief among them is that firms will quickly and efficiently adopt AI for commercial use. This should not be taken as a given. As Bessen et al. [40, 41] point out that, just because new technologies have commercial applications

does not mean that they will be adopted and diffused in a timely manner. Therefore, understanding the factors that influence the adoption and diffusion of AI in firms is important. It enables more accurate forecasting and better planning for policymakers, businesses, and civil society.

Explanatory variables for AI adoption and diffusion.

Research on the factors that affect firms' decisions to adopt digital technologies is well established [42–44]. Researchers have closely examined the adoption dynamics of innovations such as personal computers [45], the Internet [46], and social media [47]. AI builds upon these digital technologies. The factors that influence the adoption of AI by firms differ by degree but not by kind. The literature suggests eight major factors influencing AI adoption rates at the firm-level:

(1) *Competition*: McKinsey Global Institute found that the extent of rivalry within markets has the largest effect on AI adoption [48]. This is consistent with game theory [49], where the marginal propensity to adopt AI depends on the proportion of rivals that have already decided to adopt. Assuming the new technology becomes broadly diffused, then early adopters typically enjoy disproportionate rewards. However, as more firms adopt, the marginal incentive to adopt diminishes as the technology provides less competitive advantages. Therefore, laggard firms are punished with shrinking market shares [50]. These competitive forces drive adoption rates as firms jostle to assert a competitive edge and advance market share [51]. However, adoption decisions are made with imperfect information as it can be difficult to know what actions competitors are taking. Competition, therefore, can drive rapid periods of adoption growth.

(2) *Firm characteristics*: The size, income level, and industry of firms have all been shown to affect the rate that a new technology is adopted [52]. For example, larger firms, by headcount and income, typically adopt digital technologies earlier and at faster rates than smaller firms. Also, firms in Financial Services and ICT industries tend to adopt digital technologies earlier and at faster rates than firms in Agricultural and Construction industries [53]. Similarly, the AI adoption indicator we propose suggests material differences between industry categories, with highest levels of adoption in Financial & Insurance Services firms and lowest in the Agriculture Industry.

(3) *Labor force skill capabilities*: Emerging technologies, such as AI, often require specific skills [40]. The availability of workers with these skills can influence the extent of adoption and diffusion [54]. The ability to access such labor competencies, however, varies between firms, industries, and economies. The implementation of AI requires strong technical competencies. These competencies are unevenly distributed between firms, industries, and economies [41]. Therefore, the more firms are able to access relevant skilled labor, the greater the likelihood that firms will adopt AI.

(4) *Digital Maturity*: Previous research has shown that the adoption of new digital technologies often depends on the adoption of previous digital technologies [55]. For instance, broadband infrastructure supports the adoption of more sophisticated digital applications. This relationship also appears to hold for AI. According to McKinsey Global Institute, firms that have adopted and absorbed cloud infrastructure and 'web 2.0 technologies', such as mobile technologies and Customer Relationship Management (CRM) systems, are more likely to adopt AI technologies [48].

(5) *Expected return on AI investments*: Firms' perceptions of the value that a new technology can create also influences adoption rates [56]. Similarly, firms that are positive about the business use cases of AI are more likely to adopt earlier and faster [48]. Conversely, firms that are uncertain about AI use cases are slower or less likely to adopt, which delays aggregate adoption rates.

(6) *AI complements*: As with other General Purpose Technologies, the more complementary technologies are developed and implemented, the faster AI will be adopted by firms [57]. That is, the more a firm invests in one type of AI, the more likely it will invest in another. For example, a retailer that implements robotic process automation to retrieve stock is more likely to adopt computer vision to identify inventory items than a retailer that has not adopted any AI technologies. Capital investment deepens as AI is increasingly absorbed in workflows.

(7) *Regulatory effects*: Regulatory effects can be important to consider when comparing the adoption rates across economies [44, 58]. For example, it is plausible that the more stringent data protection regulations in Europe could delay AI adoption in European firms compared to US firms in the short-run.

(8) *Standardization and usability*: As the use of emerging technologies are standardized across firms and industries, the ease of use for these technologies naturally

improves, which has been shown to accelerate adoption and diffusion rates [59]. While AI models are still 'narrow' in the sense that they tend to be highly specific to a particular task and require non-routine customization (as in hyper-parameter tuning or feature data engineering), AI usability has improved over the past decade. For example, individuals are able to implement high-performing machine learning models using their own data with little or no knowledge of scripting languages (see [60]). As the use of AI technologies become standardized and usability improves, it is likely that this will increase adoption rates.

While other variables could affect rates of AI adoption, the eight factors listed above are likely to account for a significant proportion of firm-level AI adoption decisions.

S3 Appendix: Using a standardized occupation taxonomy – ANZSCO.

All data sources mentioned above correspond to their respective occupational classes according to the Australian and New Zealand Standard Classification of Occupations (ANZSCO) [61]. ANZSCO provides a basis for the standardized collection, analysis and dissemination of occupational data for Australia and New Zealand. The structure of ANZSCO has five hierarchical levels - major group(1-digit), sub-major group (2-digit), minor group (3-digit), unit group (4-digit) and occupation group (6-digit). For visualizing the distance between occupations in Fig 1.B in Skill-driven Recommendations for Job Transition Pathways, the 6-digit grouping level was applied. We used the 6-digit level here because (1) it is the most detailed occupational grouping and (2) the automation probabilities from Frey & Osborne were mapped at this level [24]. The presented results for the 'Job Transitions Recommender System' and the subset of the *Transitions Map* shown in Fig. 3 are grouped at the 4-digit unit grouping level. Occupational recommendations were made at this level to match the ground-truth of actual transitions from the Household, Income and Labour Dynamics in Australia (HILDA) longitudinal dataset [62].

Shortcomings of ANZSCO.

There are shortcomings to analyzing occupations within ANZSCO classifications. Official occupational classifications, like ANZSCO, are often static taxonomies and are rarely updated. They therefore fail to capture and adapt to emerging skills, which can misrepresent the true labor dynamics of particular jobs. For example, a ‘Data Scientist’ is a relatively new occupation that has not yet received its own ANZSCO classification. Instead, it is classified as an ‘ICT Business & Systems Analyst’ by ANZSCO, grouped with other job titles like ‘Data Analysts’, ‘Data Engineers’, and ‘IT Business Analysts’. However, as ANZSCO is the official and prevailing occupational classification system, all data used for this research are in accordance with the ANZSCO standards.

Mapping O*NET to ANZSCO.

To leverage the strengths of earlier research by Frey and Osborne [24] on the occupational risks of labor automation caused by AI technologies, we first needed to map O*NET occupations to ANZSCO, so as to take advantage of their automation risk probabilities at the 6-digit level. O*NET is a standardized and publicly available database of labor market data in the United States [63]. The occupations, however, are slightly different compared to ANZSCO. Therefore, we used a concordance table from the Australian Federal Department of Education, Skills and Employment [64] to map O*NET occupations to ANZSCO at the 6-digit level. This resulted in each ANZSCO occupation being assigned an automation risk probability according to the Frey and Osborne research.

S4 Appendix: Model Features

Below is a summary of the features included within the ‘Job Transitions Recommender System’ models. The features have been grouped into ‘Labor Demand’ (job ads data) and ‘Labor Supply’ (employment statistics) categories for ease of review. Each feature in S1 Table is measured at the ANZSCO 4-digit level per calendar year from 2012-2018. This reflects the first available year of the longitudinal job ads data (2012) and the most recent year of the ‘ground-truth’ HILDA data (2018). The ‘source’ and ‘target’ occupations are independently associated with each of the features in S1 Table.

However, the ‘theta’ (distance between skill sets measure) and ‘Difference’ features relate to both the ‘source’ and ‘target’ occupational pair. In total there are 19 features.

S1 Table Summary of constructed features and their explanation.

	Feature	Description
Labor Demand	theta:	Skills Space distance between ‘source’ and ‘target’ occupation
	Posting Frequency:	number of job advertisement vacancies
	Posting Frequency Difference:	difference between the ‘source’ and ‘target’ posting frequency
	Median Salary:	maximum median salary advertised
	Salary Difference:	difference between the ‘source’ and ‘target’ salaries
	Minimum Education:	minimum years of formal education required
	Education Difference:	difference between the ‘source’ and ‘target’ years of formal education required
	Minimum Experience:	minimum years of formal experience required
	Experience Difference:	difference between the ‘source’ and ‘target’ years of experience required
Labour Supply	Total Employed:	total number employed at ANZSCO Unit level (000’s)
	Total Employed Difference:	difference between the ‘source’ and ‘target’ of total number employed at ANZSCO Unit level (000’s)
	Total Hours Worked:	total hours worked at ANZSCO Unit level (000’s)
	Total Hours Worked Difference:	difference between the ‘source’ and ‘target’ of total hours worked at ANZSCO Unit level (000’s)

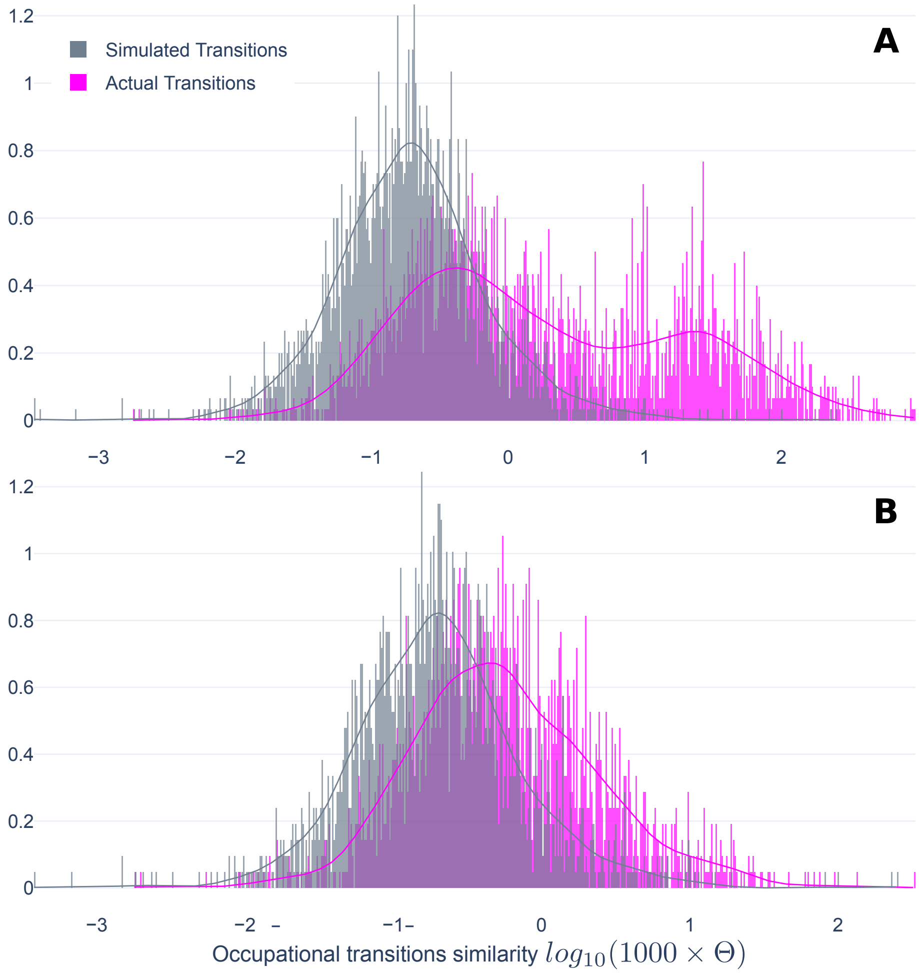

Fig 1. Density plots for the statistical tests against all occupational transitions (A) and against transitions where the worker changed occupations (B).

S5 Appendix: Validation

Statistical Test.

To obtain initial validation of the Skills Space distance measures, we conducted a paired statistical test, as explained in Skill-driven Recommendations for Job Transition Pathways. To run this experiment, we labeled each 'source' and 'target' occupational pair with their distance measure for their given year (called the 'True Sample'). We then simulated an alternate sample of transitions where we maintain the same 'source' occupations and randomly select 'target' occupations, all assigned with their pairwise distance scores (called 'Simulated Sample'). Fig. 1-A shows the distribution of all job transitions, including transitions to the same occupation. We found that the differences between the 'True' and 'Simulated' transition samples are statistically significant (t-statistic = 16.272, p-value = 2.707×10^{-58}, Cohen's D effect size = 0.42). However, 909 of the 2999 (or 30%) of the yearly job transitions from 2012-2018 are movements to the same occupation. Intuitively, the skill set distance of transitioning to another job in the same occupation is likely small, especially compared to other occupations. Therefore, we wanted to test whether the statistical significance holds when we exclude transitions to the same occupation. To run this test, we first removed job transitions to the same occupation (leaving 2090 occupations from 2012-2018). Following the same process described above, we created 'True' and 'Simulated' samples. Fig. 1-B illustrates the differences between the two samples, which are again statistically significant (t-statistic = 4.514, p-value = 6.535×10^{-06}); however, the effect size is lowered (Cohen's D effect size = 0.14).

We repeated the procedure 100 times: we generated 100 'Random' populations and we perform the statistical testing for each. As shown in Skill-driven Recommendations for Job Transition Pathways, 87 of the 100 obtained p-values were lower than 0.05 providing confidence in the statistical significance of Skills Space to represent occupational transitions in this research.

Job Transitions Recommender System validation.

Fig. 2-A shows the confusion matrix for the binary classifier model containing all of the features from S1 Table. This feature configuration achieved the highest performance

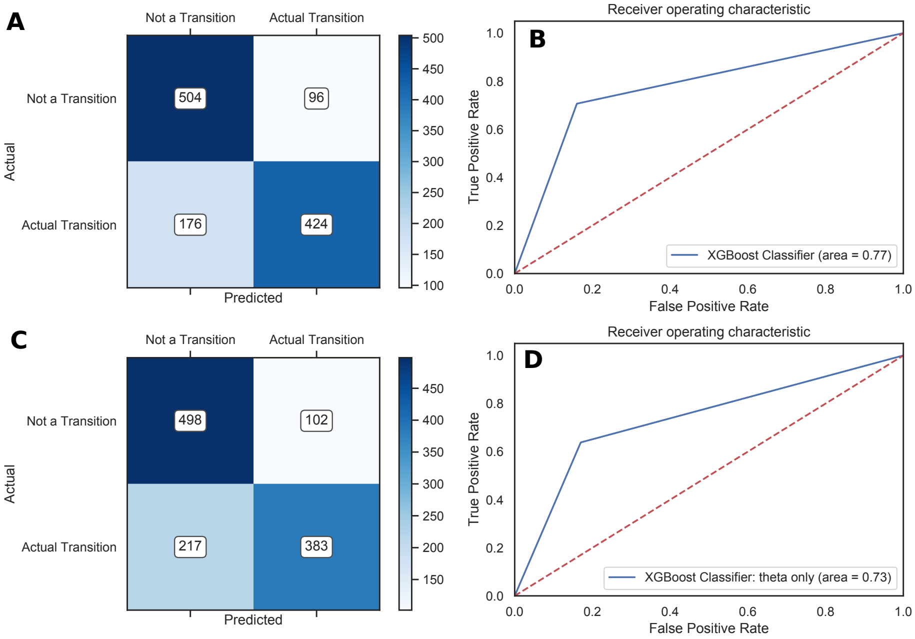

Fig 2. Prediction performance and confusion matrix. Job transition model that includes all features achieves the highest results, as seen with (A) the confusion matrix and (B) the ROC curve; whereas the job transitions classifier model that only includes SKILLS SPACE distance method has lower performance, as seen by (C) the confusion matrix and (D) the ROC curve.

(Accuracy = 76% and F1 Macro Average = 77%). As observed, this trained model was able to predict *True Negatives* ('Not a Transition' – Recall = 84%) slightly better than *True Positives* ('Actual Transition' – Recall = 71%). Fig. 2-B shows the 'receiver operating characteristic' curve (ROC curve), which is the performance of the binary classification model at all classification thresholds. ROC curves summarize the trade-off between the *True Positive* rate (y-axis) and *False Positive* rate (x-axis) for the classifier model using different probability thresholds. Generally, high-performing models are represented by ROC curves that bow up to the top left of the plot. As illustrated in Fig. 2-B, the blue ROC curve is consistently above the diagonal red dashed line that represents a 50% probability – models that perform below this dashed line are no better than random chance. This reinforces that the model consisting of 'All Features' achieves strong performance levels.

Similarly, Fig. 2-C shows the confusion matrix and Fig. 2-D illustrates the ROC curve for the classifier model that only includes the SKILLS SPACE distance measure ('theta'). While the 'theta only' model still performs relatively well (Accuracy & F1 Macro Average = 73%), performance does decline. Again, the *True Negatives* ('Not a

Transition' – Recall = 83%) outperform the *True Positives* ('Actual Transition' – Recall = 64%). This highlights that the added labor market features from job ads data and employment statistics increased the performance capabilities of the models to predict *True Positives*. These performance differences are also represented in Fig. 2-D showing a slightly lower ROC curve for models that used 'theta' alone.

Ablation Test and Feature Importance.

In order to understand the relative importance of the modeled features in the 'Job Transitions Recommender System', we conducted an ablation test and feature importance analysis. An ablation test involves iteratively removing one feature from the feature set and then re-training the model to make predictions and evaluate performance. We conclude that a feature is 'more important' to a model's predictive capabilities if performance declines when it is removed. Fig. 3-A shows the results of all 19 features, highlighting that the largest performance decline occurred when the 'theta' distance measure was removed. These models were all trained with a consistent setup, as explained in Skill-driven Recommendations for Job Transition Pathways.

To reinforce the results from the ablation test, we then conducted a feature importance analysis as seen below. We use the 'Gain' metric, which shows the relative contribution of each feature to the model by calculating the features' contribution for each tree in the XGBoost model. A higher gain score indicates that a feature is more important for generating a prediction. Again, 'theta' is overwhelmingly identified as the most important feature for predicting job transitions.

S6 Appendix: Recommending Jobs & Skills

S2 Table Transitions Example – Domestic Cleaner

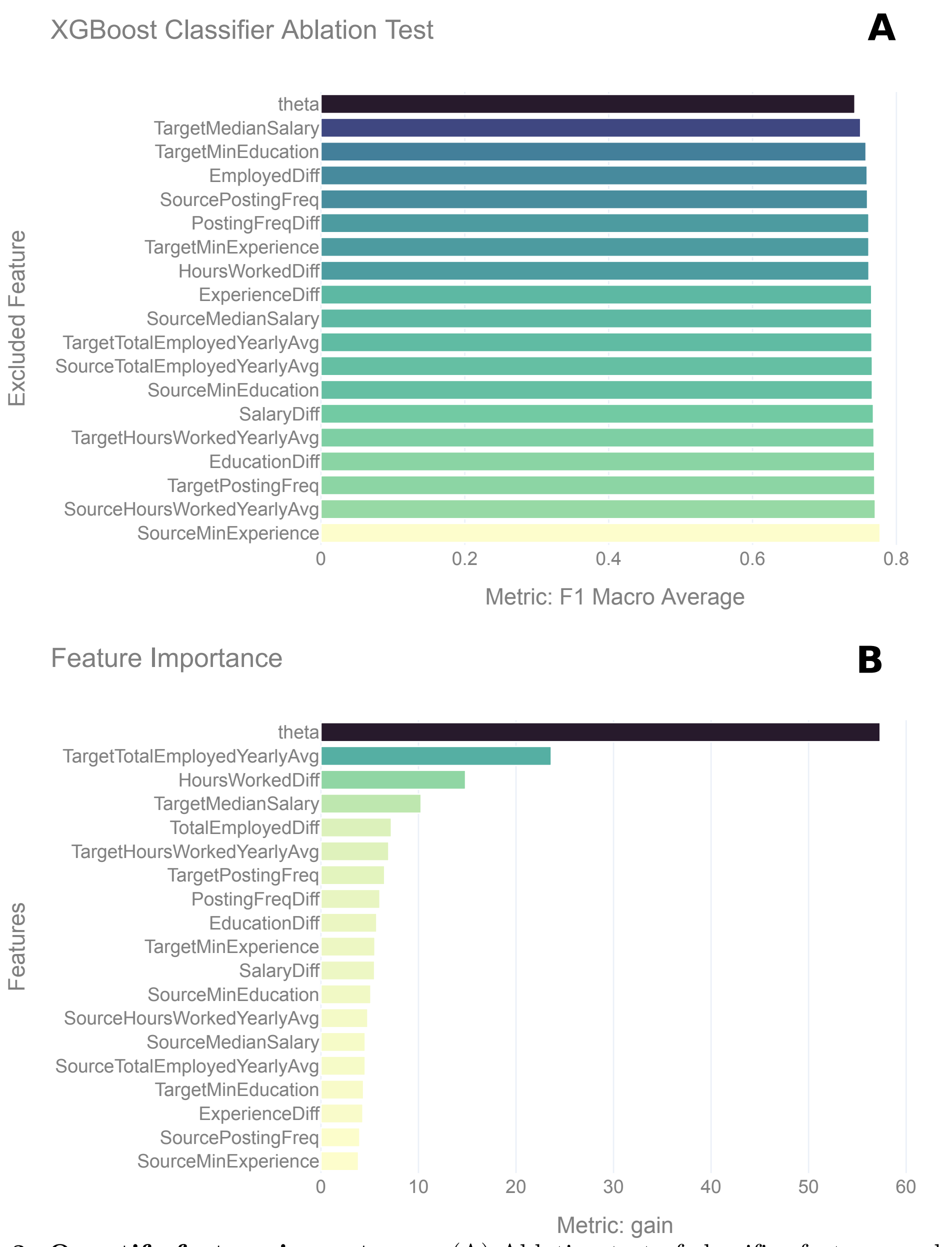

Fig 3. Quantify feature importance. (A) Ablation test of classifier features and (B) feature importance analysis both show that the SKILLS SPACE distance measure (‘theta’) is the most important feature for predicting occupational transitions.

Occupation	Transition Probability	Num. Job Ads 2019	Num. Job Ads 2020	Difference	Percentage Difference
Domestic Cleaners	0.960395	323	276	-47	-14.551084
Commercial Cleaners	0.946621	865	671	-194	-22.427746
Waiters	0.943874	690	253	-437	-63.333333
Bar Attendants and Baristas	0.937961	600	180	-420	-70.000000
Sales Assistants (General)	0.935315	2835	1609	-1226	-43.245150
Chefs	0.926472	1904	877	-1027	-53.939076
Cooks	0.914349	726	356	-370	-50.964187
Aged and Disabled Carers	0.893725	961	1302	341	35.483871
Child Carers	0.887601	837	414	-423	-50.537634
General Clerks	0.876921	2281	1466	-815	-35.729943

S7 Appendix: AI Adoption

S3 Table AI Similarity Scores. The table below contains the underlying data for the AI Adoption radar chart in the *Developing a Leading Indicator of AI Adoption* section.

Industry	2013	2016	2019	Percentage change 13-19
Financial and Insurance Services	0.000958	0.001599	0.002887	201.395294
Information Media and Telecommunications	0.001057	0.001283	0.002286	116.285278
Professional, Scientific and Technical Services	0.000545	0.001027	0.001590	191.537693
Retail Trade	0.000266	0.000568	0.001348	407.375732
Electricity, Gas, Water and Waste Services	0.000443	0.000520	0.001300	193.594618
Education and Training	0.000707	0.000933	0.001257	77.744881
Transport, Postal and Warehousing	0.000282	0.000427	0.000981	247.957226
Public Admin	0.000270	0.000481	0.000905	234.594611
Rental, Hiring and Real Estate Services	0.000157	0.000439	0.000775	392.117595
Arts and Recreation Services	0.000256	0.000571	0.000738	188.519596
Manufacturing	0.000206	0.000356	0.000690	235.589203
Administrative and Support Services	0.000243	0.000346	0.000661	172.344264
Wholesale Trade	0.000219	0.000388	0.000642	193.307221
Mining	0.000260	0.000223	0.000595	128.900314
Construction	0.000136	0.000236	0.000487	257.990332
Other Services	0.000136	0.000180	0.000306	124.906123
Health Care and Social Assistance	0.000080	0.000170	0.000281	252.858476
Accommodation and Food Services	0.000086	0.000208	0.000267	208.987030
Agriculture, Forestry and Fishing	0.000108	0.000183	0.000239	120.979250

Temporal AI skill similarity to Australian Industries. The figure below is another visualization of the same data in the *Developing a Leading Indicator of AI*

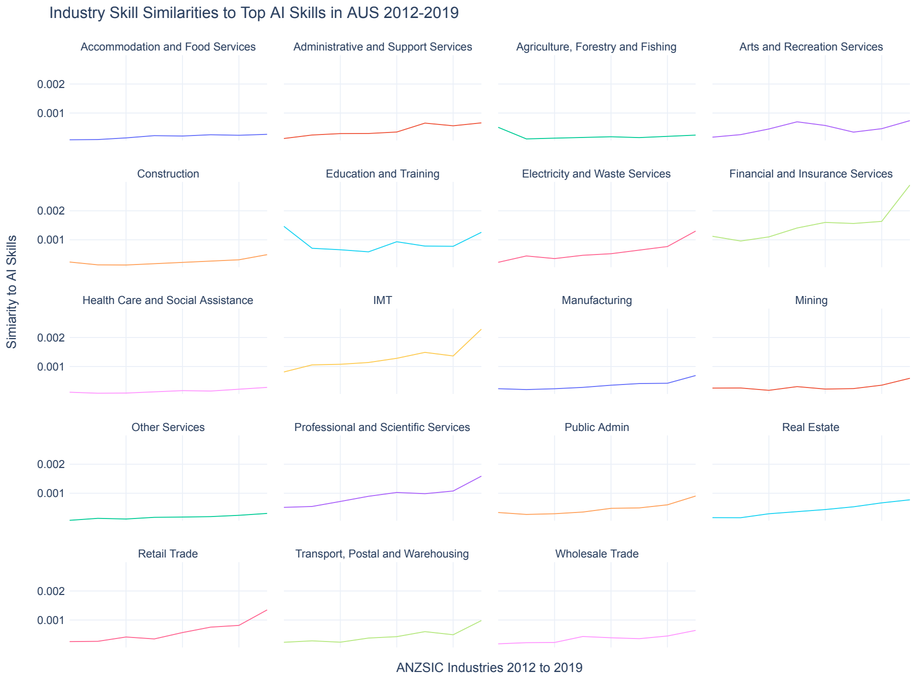

Fig 4. Yearly skill similarities between AI skills and Australian Industry (ANZSIC Division) skill sets from 2012-2019.

Adoption section illustrating all of the yearly AI similarity scores for the Industries from 2012-2019.

S8 Appendix: Skill Count Distribution

As discussed in *Materials & Methods*, before calculating individual skill similarities, we filtered out extremely rare skills to reduce noise and computational complexity. We set the minimum yearly skill count threshold to be greater than or equal to 5.

As seen in the Empirical Cumulative Distribution Function (ECDF) in Fig. 5, this threshold represents over 75% of all skills in 2018 (6,981 skills). All skills on the left side of the dotted threshold line were excluded, which accounted for <25% of skills.

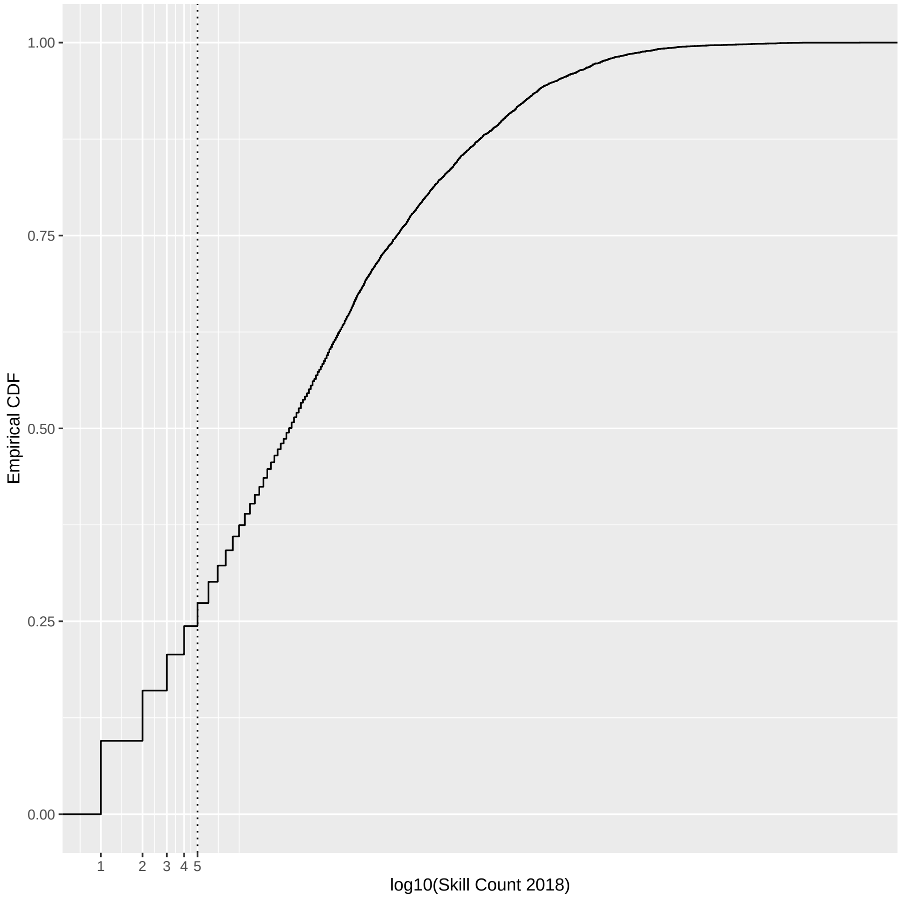

Fig 5. Empirical Cumulative Distribution Function of skill counts within job ads for 2018.

S9 Appendix: Posting Frequency of AI Seed Skills

We selected five ‘seed skills’ to construct a dynamic list of yearly AI skills, as described in Sec. *Materials & Methods* and visualized in Fig. 6 in the main paper. This allowed us to measure the distance between AI skills and industry skill sets, capturing both the evolution of skill demands and accounting for skill importance. The most common method, however, is to simply count the frequency of a skill (or group of skills) over time. In Fig. 6, we show the posting frequency of the five AI seed skills used to construct the dynamic list of yearly AI skills. The five AI seed skills being: (1) Artificial Intelligence; (2) Machine Learning; (3) Data Science; (4) Data Mining; and (5) Big Data.

As Fig. 6 shows, the posting frequency for all five seed skills increases from 2012 to 2019, albeit at different rates. ‘Data Science’ experiences the steepest increases over this period. Whereas ‘Data Mining’ has had more modest growth, reaching its highest posting frequency levels in 2015 and has since declined.

Fig. 7 shows that not only have the absolute posting frequencies of the AI seed skills increased, but also the percentage of vacancies containing these skills. In 2012, approximately 0.13% of Australian job ads (or 783 vacancies) contained at least one of the AI seed skills. In 2019, this had increased to more than 1.3% of job ads (or 13,399 vacancies) – growth of over ten times the percentage of job ads requiring the AI seed skills.

While these simple metrics provide an indication of the degree of growth required by the AI seed skills, there are some fundamental shortcomings for using posting frequencies as a proxy for labor demand. A discussion on the disadvantages of using posting frequency compared to skill similarity follows.

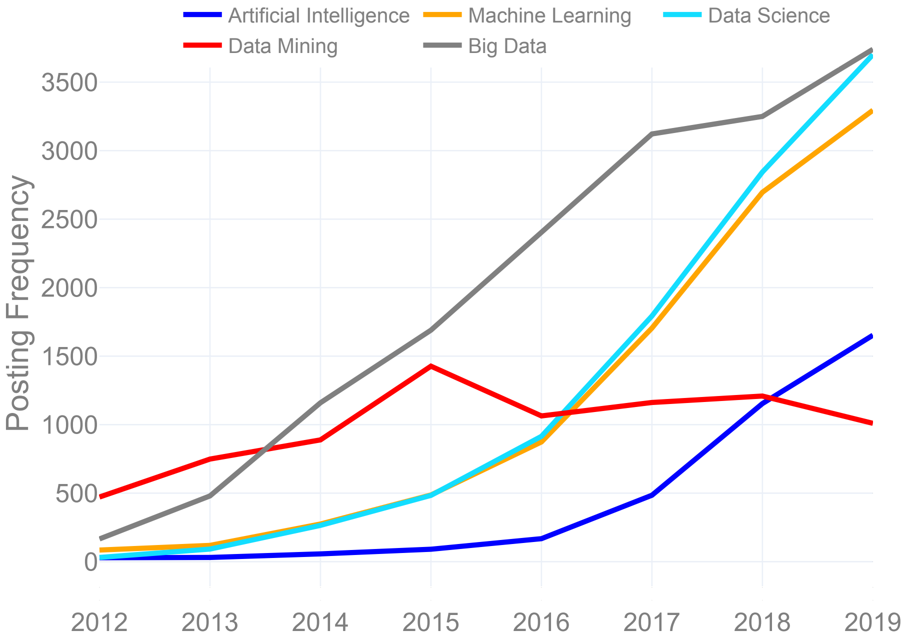

Fig 6. Posting frequency of AI skills. Yearly posting frequency of the five AI seed skills used to build a dynamic list of yearly AI skills.

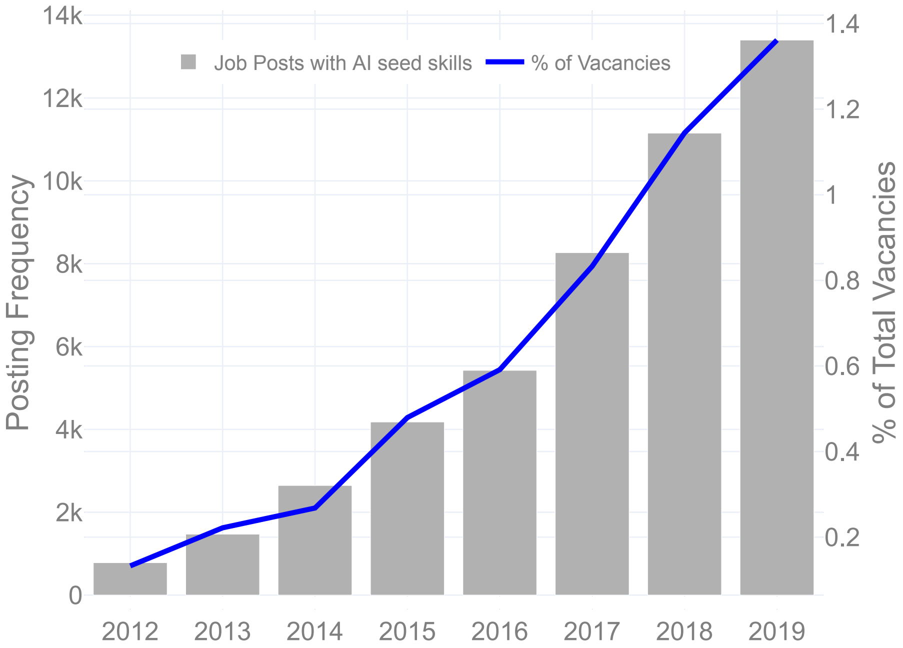

Fig 7. Vacancy rate of AI skills. The percentage of vacancies in Australia that contain these five AI seed skills.

S10 Appendix: Advantages of Skill Similarity over Posting Frequency

The proxy most widely used in literature [65] for skill importance is skill frequency. This simply counts how many times a skill appears in job ads associated with a given occupation (or other groups) during a predetermined period of time; the higher the count, the greater the demand and, implicitly, the greater importance of the skill to the occupation. While skill frequency can provide some indication of labor demand, it fails to normalize for skills that are demanded by all or most jobs. This does not necessarily reveal which skills are more or less important to a given occupation, as some skills generalize across all occupations at high frequencies. For example, 'Communication Skills' and 'Teamwork' occur in over one-quarter of all job ads and are ubiquitous across all occupations). However, we know that some skills are more important than others to specific jobs. We therefore capture a proxy for skill importance by measuring the comparative advantage of each skill in each job ad, as seen in the RCA equation in *Materials & Methods*. Our measure controls for high-occurring skills through normalization and develops a measure of skill importance within individual job ads that later represent skill importance within labor market groups (occupations, industries etc.).

References

1. Borjas GJ, Van Ours JC. Labor economics. McGraw-Hill/Irwin Boston; 2010.
2. Schultz TW. Investment in Human Capital. The American Economic Review. 1961;51(1):1–17.
3. Becker G. Human capital. Columbia University: Columbia University Press; 1964.
4. Becker GS, Murphy KM, Tamura R. Human Capital, Fertility, and Economic Growth. Journal of Political Economy. 1990;98(5, Part 2):S12–S37.
5. Pries M, Rogerson R. Hiring policies, labor market institutions, and labor market flows. Journal of Political Economy. 2005;113(4):811–839.

6. Bassanini A, Garnero A. Dismissal protection and worker flows in OECD countries: Evidence from cross-country/cross-industry data. Labour Economics. 2013;21:25–41.

7. Hassler J, Rodriguez Mora JV, Storesletten K, Zilibotti F. A positive theory of geographic mobility and social insurance. International Economic Review. 2005;46(1):263–303.

8. Goldin CD. In: Human Capital. Heidelberg, Germany: Springer Verlag; 2016.

9. Nedelkoska L, Neffke F. Skill Mismatch and Skill Transferability: Review of Concepts and Measurements. Papers in Evolutionary Economic Geography. 2019;.

10. Wasmer E. General versus specific skills in labor markets with search frictions and firing costs. American Economic Review. 2006;96(3):811–831.

11. OECD. OECD Skills Strategy 2019 - Skills to Shape a Better Future. OECD; 2019.

12. Gardiner A, Aasheim C, Rutner P, Williams S. Skill Requirements in Big Data: A Content Analysis of Job Advertisements. Journal of Computer Information Systems. 2018;58(4):374–384.

13. Topel RH, Ward MP. Job mobility and the careers of young men. The Quarterly Journal of Economics. 1992;107(2):439–479.

14. Freeman RB. Overinvestment in college training? Journal of human resources. 1975; p. 287–311.

15. Goldin CD, Katz LF. The race between education and technology. Harvard University Press; 2009.

16. Vona F, Consoli D. Innovation and skill dynamics: a life-cycle approach. Industrial and Corporate Change. 2015;24(6):1393–1415.

17. Mincer J. Human capital, technology, and the wage structure: what do time series show? National Bureau of Economic Research; 1991.

18. Berman E, Bound J, Griliches Z. Changes in the demand for skilled labor within US manufacturing: evidence from the annual survey of manufactures. The Quarterly Journal of Economics. 1994;109(2):367–397.

19. Autor DH, Katz LF, Krueger AB. Computing inequality: have computers changed the labor market? The Quarterly journal of economics. 1998;113(4):1169–1213.

20. Acemoglu D, Autor D. Skills, tasks and technologies: Implications for employment and earnings. In: Handbook of Labor Economics. vol. 4. Elsevier; 2011. p. 1043–1171.

21. Autor DH, Handel MJ. Putting tasks to the test: Human capital, job tasks, and wages. Journal of labor Economics. 2013;31(S1):S59–S96.

22. Goos M, Manning A, Salomons A. Explaining job polarization: Routine-biased technological change and offshoring. American economic review. 2014;104(8):2509–26.

23. Brynjolfsson E, McAfee A. The second machine age: Work, progress, and prosperity in a time of brilliant technologies. WW Norton & Company; 2014.

24. Frey CB, Osborne MA. The Future of Employment: How susceptible are jobs to computerisation? Technological Forecasting and Social Change. 2017;114:254–280.

25. Brown TB, Mann B, Ryder N, Subbiah M, Kaplan J, Dhariwal P, et al. Language Models are Few-Shot Learners. In: Advances in Neural Information Processing Systems (NeurIPS 2020); 2020.

26. Touvron H, Vedaldi A, Douze M, Jégou H. Fixing the train-test resolution discrepancy: FixEfficientNet; 2020.

27. Silver D, Hubert T, Schrittwieser J, Antonoglou I, Lai M, Guez A, et al. A general reinforcement learning algorithm that masters chess, shogi, and Go through self-play. Science. 2018;362(6419):1140–1144.

28. Blinder AS, Krueger AB. Alternative measures of offshorability: a survey approach. Journal of Labor Economics. 2013;31(S1):S97–S128.

29. Autor DH, Dorn D, Hanson GH. The China Syndrome: Local Labor Market Effects of Import Competition in the United States. Am Econ Rev. 2013;103(6):2121–2168.

30. Shaw KL. A formulation of the earnings function using the concept of occupational investment. Journal of Human Resources. 1984; p. 319–340.

31. Shaw KL. Occupational change, employer change, and the transferability of skills. Southern Economic Journal. 1987; p. 702–719.

32. Poletaev M, Robinson C. Human capital specificity: evidence from the Dictionary of Occupational Titles and Displaced Worker Surveys, 1984–2000. Journal of Labor Economics. 2008;26(3):387–420.

33. Ingram BF, Neumann GR. The returns to skill. Labour economics. 2006;13(1):35–59.

34. Gathmann C, Schönberg U. How general is human capital? A task-based approach. Journal of Labor Economics. 2010;28(1):1–49.

35. Alabdulkareem A, Frank MR, Sun L, AlShebli B, Hidalgo C, Rahwan I. Unpacking the polarization of workplace skills. Science Advances. 2018;4(7):eaao6030.

36. Dawson N, Rizoiu MA, Johnston B, Williams MA. Adaptively selecting occupations to detect skill shortages from online job ads. In: 2019 IEEE International Conference on Big Data (Big Data). IEEE; 2019. p. 1637–1643.

37. Nedelkoska L, Neffke F, Wiederhold S. Skill mismatch and the costs of job displacement. In: Annual Meeting of the American Economic Association; 2015.

38. Bechichi N, Grundke R, Jamet S, Squicciarini M. Moving between jobs; 2018.

39. Grundke R, Jamet S, Kalamova M, Squicciarini M. Having the right mix: The role of skill bundles for comparative advantage and industry performance in GVCs; 2017.

40. Bessen J. Technology adoption costs and productivity growth: The transition to information technology. Review of Economic Dynamics. 2002;.

41. Bessen JE, Impink SM, Seamans R, Reichensperger L. The Business of AI Startups; 2018.

42. Rogers EM. New product adoption and diffusion. Journal of consumer Research. 1976;2(4):290–301.

43. Karahanna E, Straub DW, Chervany NL. Information technology adoption across time: a cross-sectional comparison of pre-adoption and post-adoption beliefs. MIS quarterly. 1999; p. 183–213.

44. Im I, Hong S, Kang MS. An international comparison of technology adoption: Testing the UTAUT model. Information & management. 2011;48(1):1–8.

45. Thong JY. An integrated model of information systems adoption in small businesses. Journal of management information systems. 1999;15(4):187–214.

46. Andrés L, Cuberes D, Diouf M, Serebrisky T. The diffusion of the Internet: A cross-country analysis. Telecommunications policy. 2010;34(5-6):323–340.

47. Perrin A. Social media usage. Pew research center. 2015; p. 52–68.

48. Bughin J, Seong J, Manyika J, Chui M, Joshi R. Notes from the AI frontier: Modeling the impact of AI on the world economy. McKinsey Global Institute; 2018.

49. Moorthy KS. Using game theory to model competition. Journal of Marketing Research. 1985;22(3):262–282.

50. Andrews D, Criscuolo C, Gal PN. Frontier firms, technology diffusion and public policy: Micro evidence from OECD countries. OECD; 2015.

51. Mamer JW, McCardle KF. Uncertainty, Competition, and the Adoption of New Technology. Management Science. 1987;33(2):161–177.

52. Hall BH, Khan B. Adoption of new technology. National bureau of economic research; 2003.

53. Business Use of Information Technology; 2017. https://www.abs.gov.au/statistics/industry/technology-and-innovation/business-use-information-technology/latest-release.

54. Beaudry P, Doms M, Lewis E. Endogenous skill bias in technology adoption: City-level evidence from the IT revolution. National Bureau of Economic Research; 2006.

55. Andrews D, Nicoletti G, Timiliotis C. Digital technology diffusion: A matter of capabilities, incentives or both? OECD; 2018.

56. Anderson ST, Newell RG. Information programs for technology adoption: the case of energy-efficiency audits. Resource and Energy economics. 2004;26(1):27–50.

57. Brynjolfsson E, Rock D, Syverson C. Artificial Intelligence and the Modern Productivity Paradox: A Clash of Expectations and Statistics. In: The Economics of Artificial Intelligence: An Agenda. University of Chicago Press; 2018.

58. Perino G, Requate T. Does more stringent environmental regulation induce or reduce technology adoption? When the rate of technology adoption is inverted U-shaped. Journal of Environmental Economics and Management. 2012;64(3):456–467.

59. Bessen J. Learning by Doing: The Real Connection between Innovation, Wages, and Wealth. Yale University Press; 2015.

60. Cloud AutoML;. https://cloud.google.com/automl.

61. Australian Bureau of Statistics. 1220.0 - ANZSCO – Australian and New Zealand Standard Classification of Occupations, 2013, Version 1.2; 2013. https://www.abs.gov.au/ausstats/abs@.nsf/0/E3031B89999B4582CA2575DF002DA702?opendocument#:~:text=The%20structure%20of%20ANZSCO%20has,grouped%20into%20'minor%20groups'.

62. Department of Social Services and Melbourne Institute of Applied Economic and Social Research. The Household, Income and Labour Dynamics in Australia (HILDA) Survey, RESTRICTED RELEASE 18 (Waves 1-18); 2020.

63. U S Department of Labor. O*NET; 2020. https://www.onetonline.org/.

64. Australian Federal Department of Education, Skills and Employment. ANZSCO to O*NET concordance;.

65. Carnevale A, Jayasundera T, Repnikov D. Understanding Online Job Ads Data. Georgetown University; 2014.
