# Supplementary figures and images for: Skill-driven recommendations for job transition pathways
Source: PLoS One. 2021 Aug 4;16(8):e0254722. doi: 10.1371/journal.pone.0254722 (PMC8336878; doi:10.1371/journal.pone.0254722)

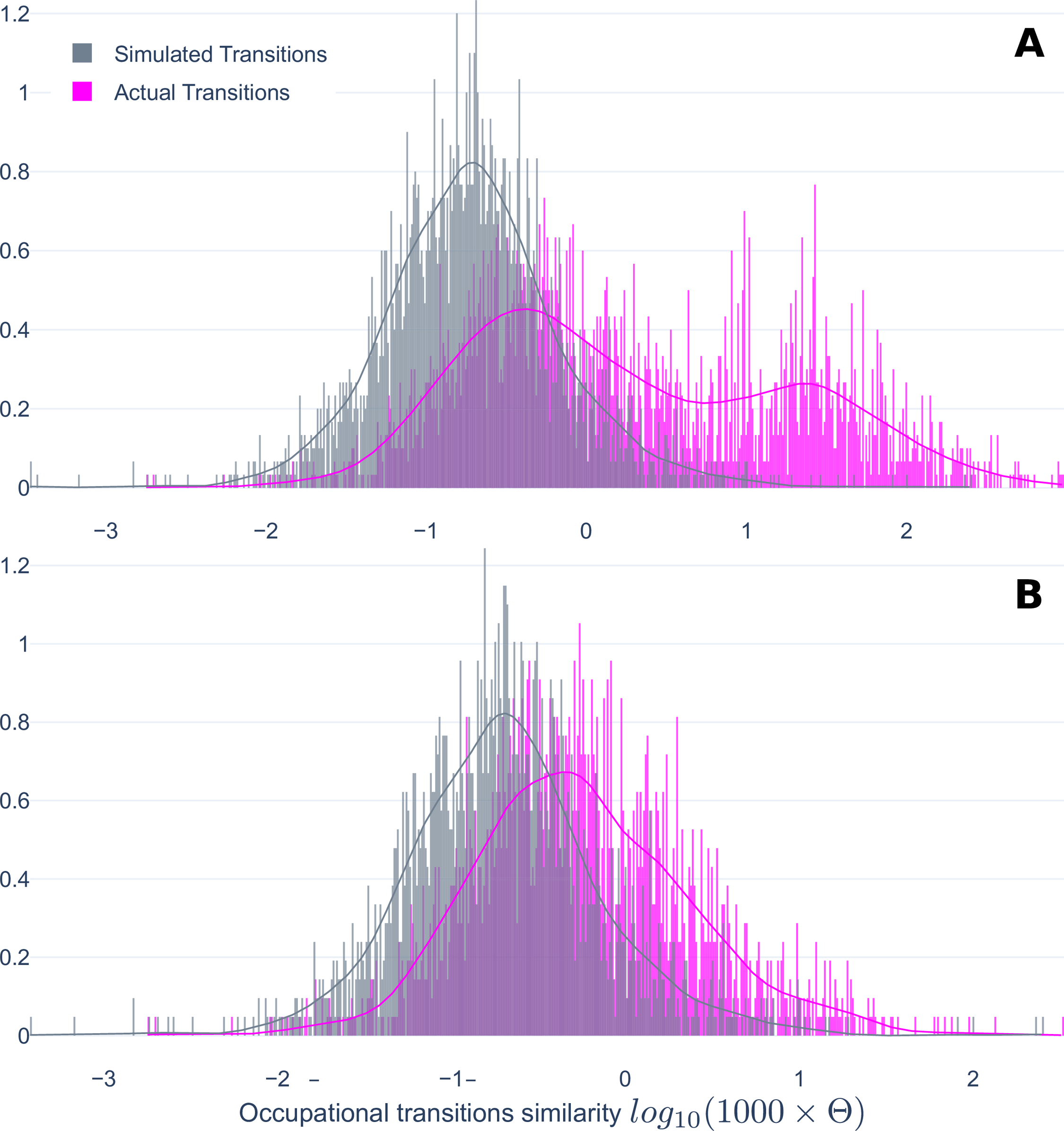

Supplement: S1 Fig — (TIFF) [file pone.0254722.s002.tiff]

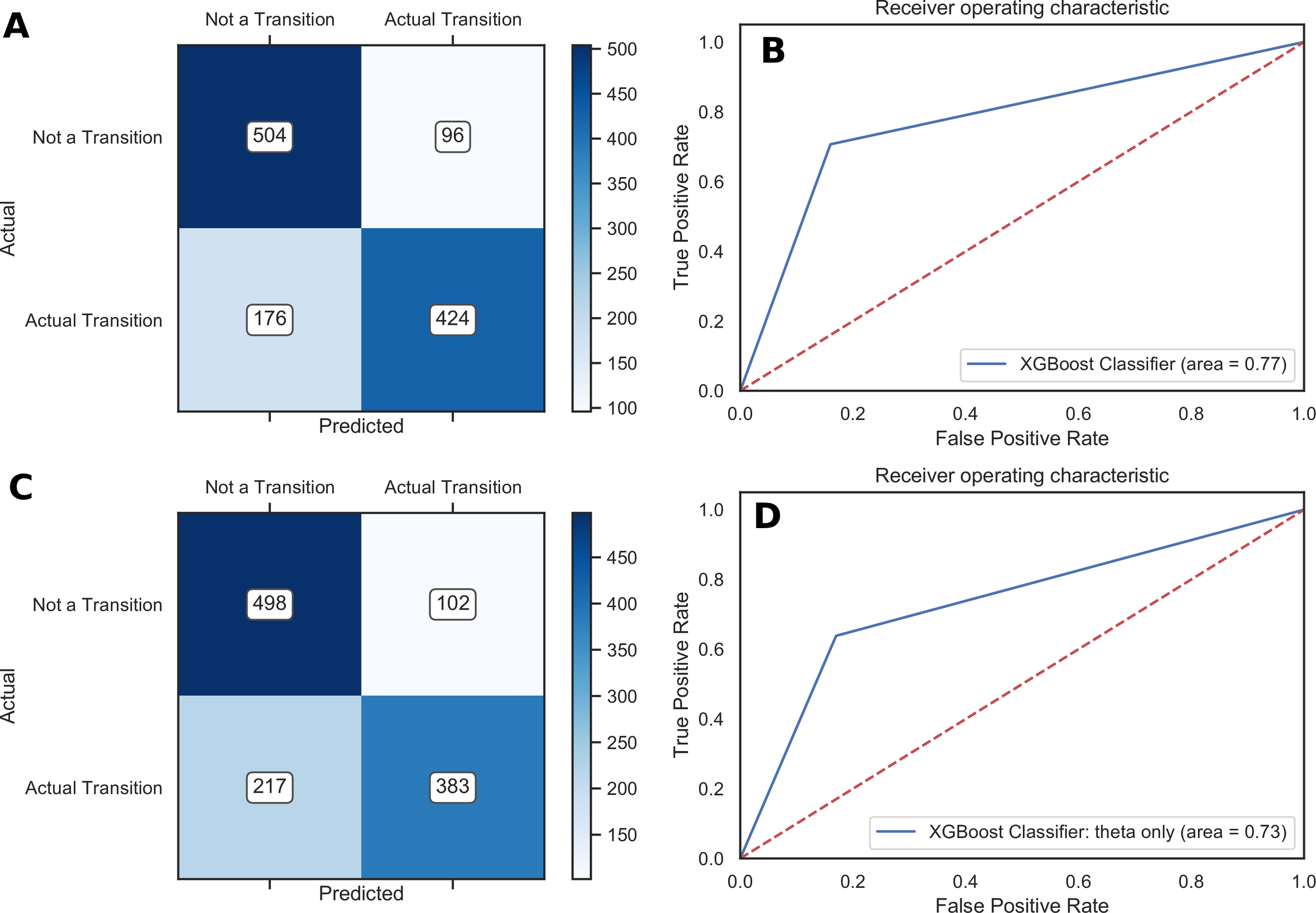

Supplement: S2 Fig — Job transition model that includes all features achieves the highest results, as seen with (A) the confusion matrix and (B) the ROC curve; whereas the job transitions classifier model that only includes Skills Space distance method has lower performance, as seen by (C) the confusion matrix and (D) the ROC curve. (TIFF) [file pone.0254722.s003.tiff]

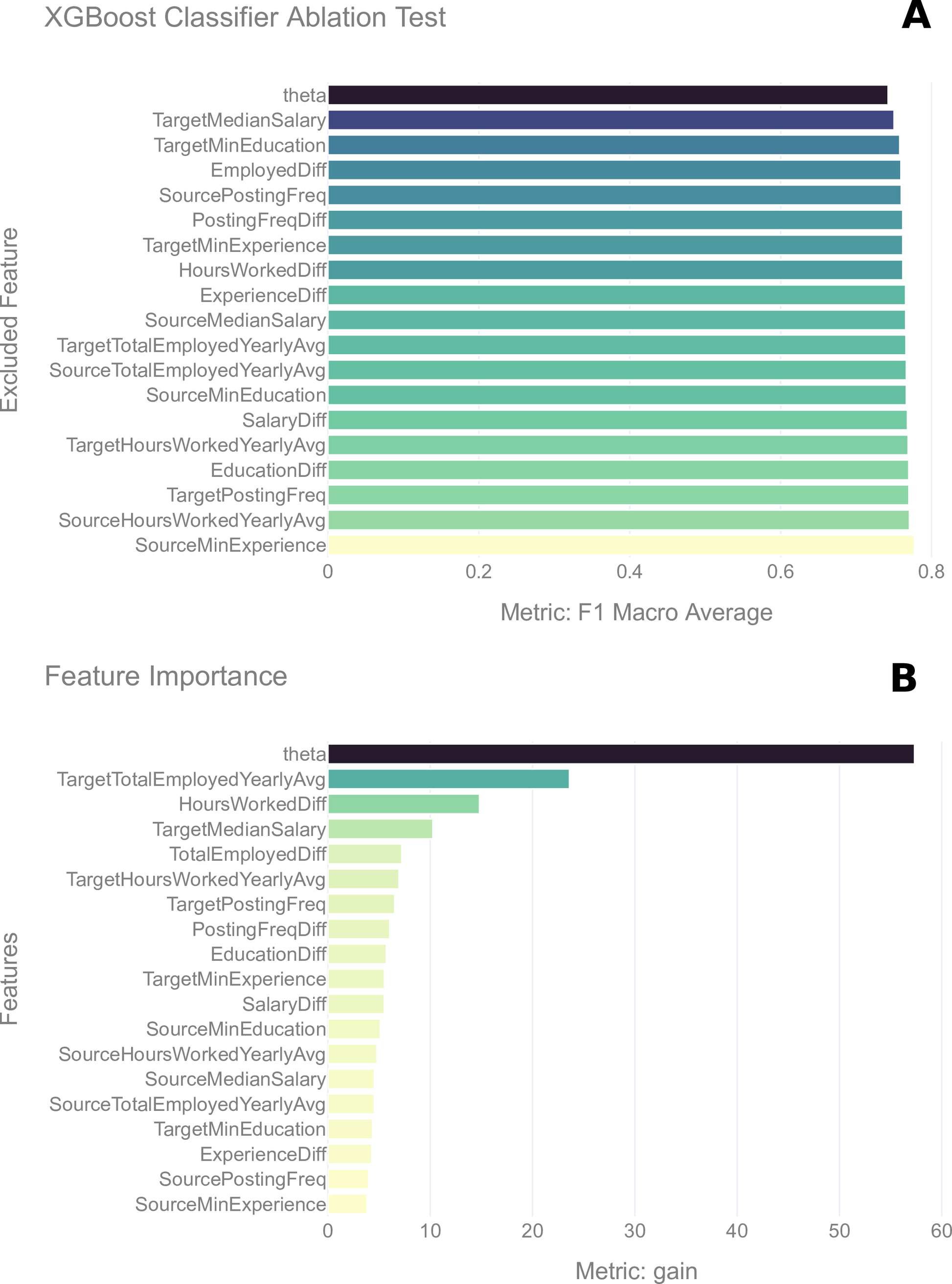

Supplement: S3 Fig — (A) Ablation test of classifier features and (B) feature importance analysis both show that the Skills Space distance measure (‘theta’) is the most important feature for predicting occupational transitions. (TIFF) [file pone.0254722.s004.tiff]

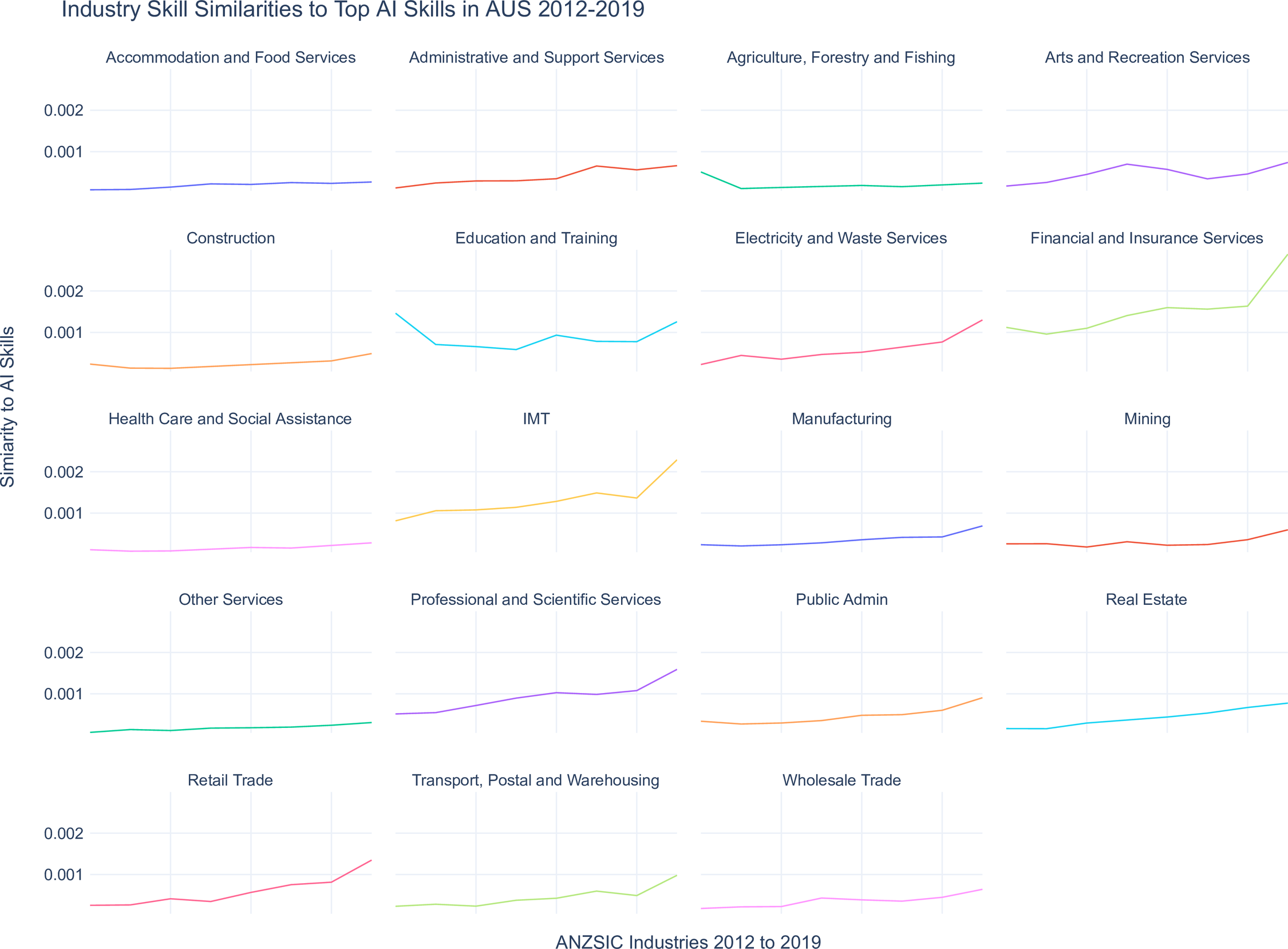

Supplement: S4 Fig — (TIFF) [file pone.0254722.s005.tiff]

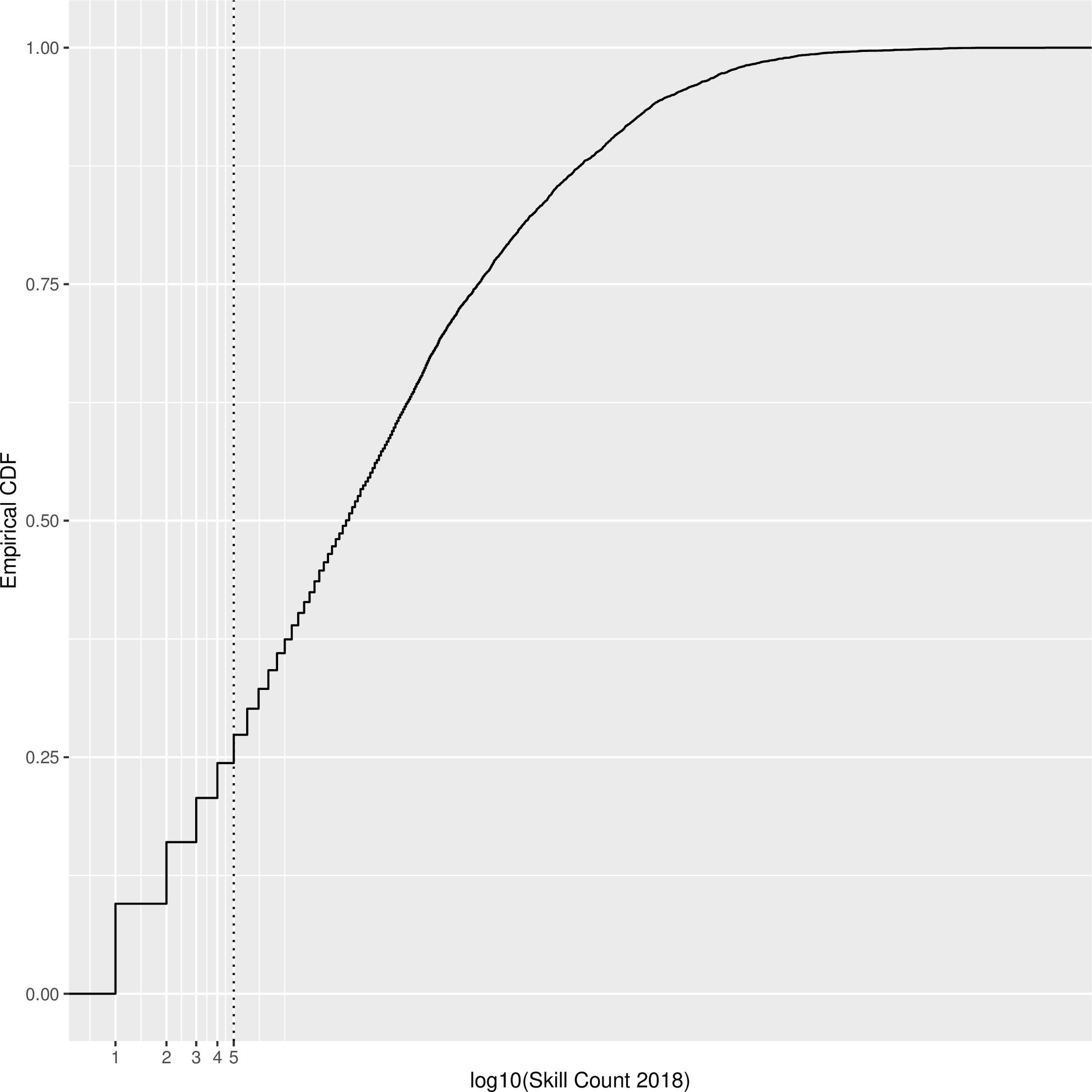

Supplement: S5 Fig — (TIFF) [file pone.0254722.s006.tiff]

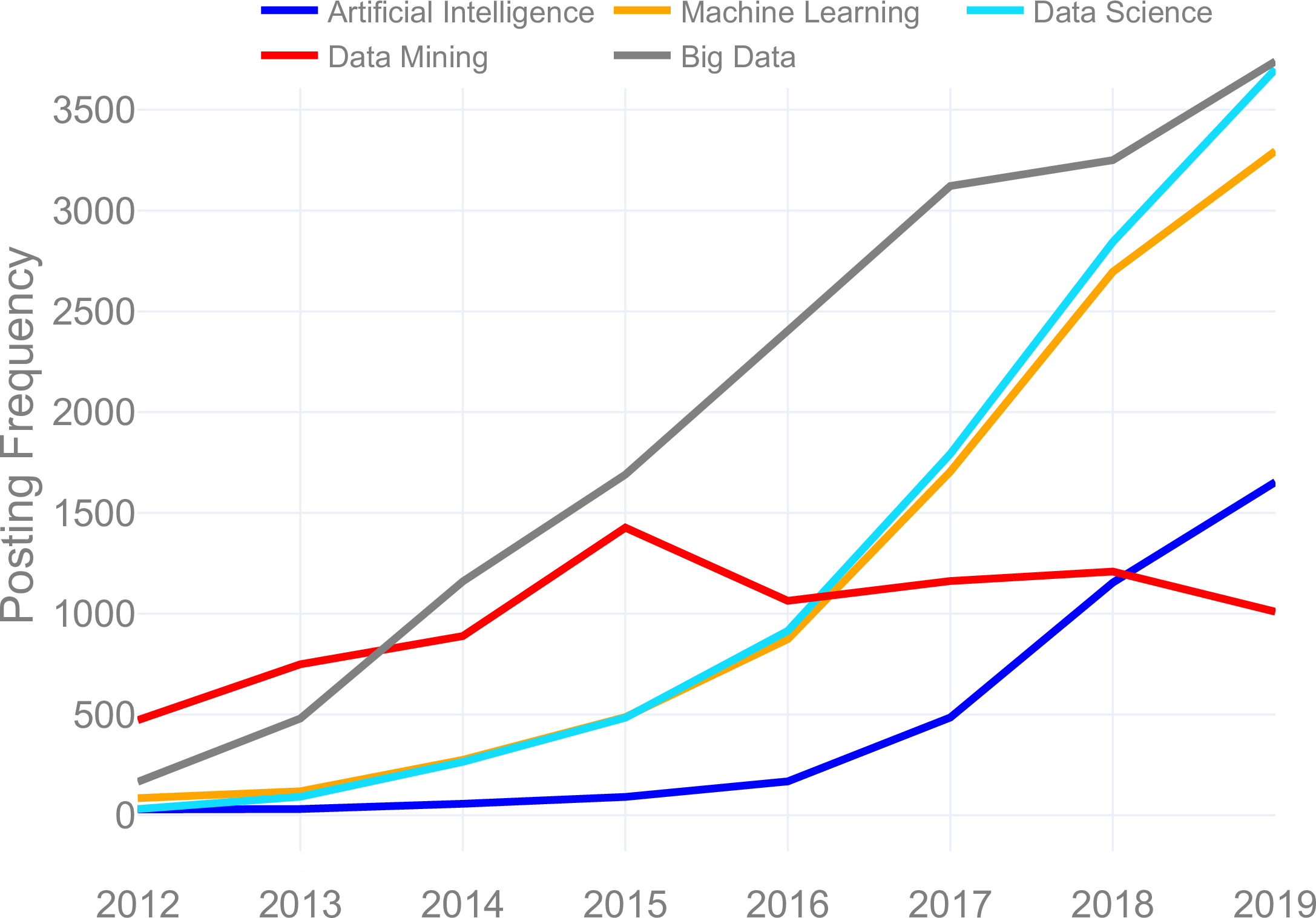

Supplement: S6 Fig — Yearly posting frequency of the five AI seed skills used to build a dynamic list of yearly AI skills. (TIFF) [file pone.0254722.s007.tiff]

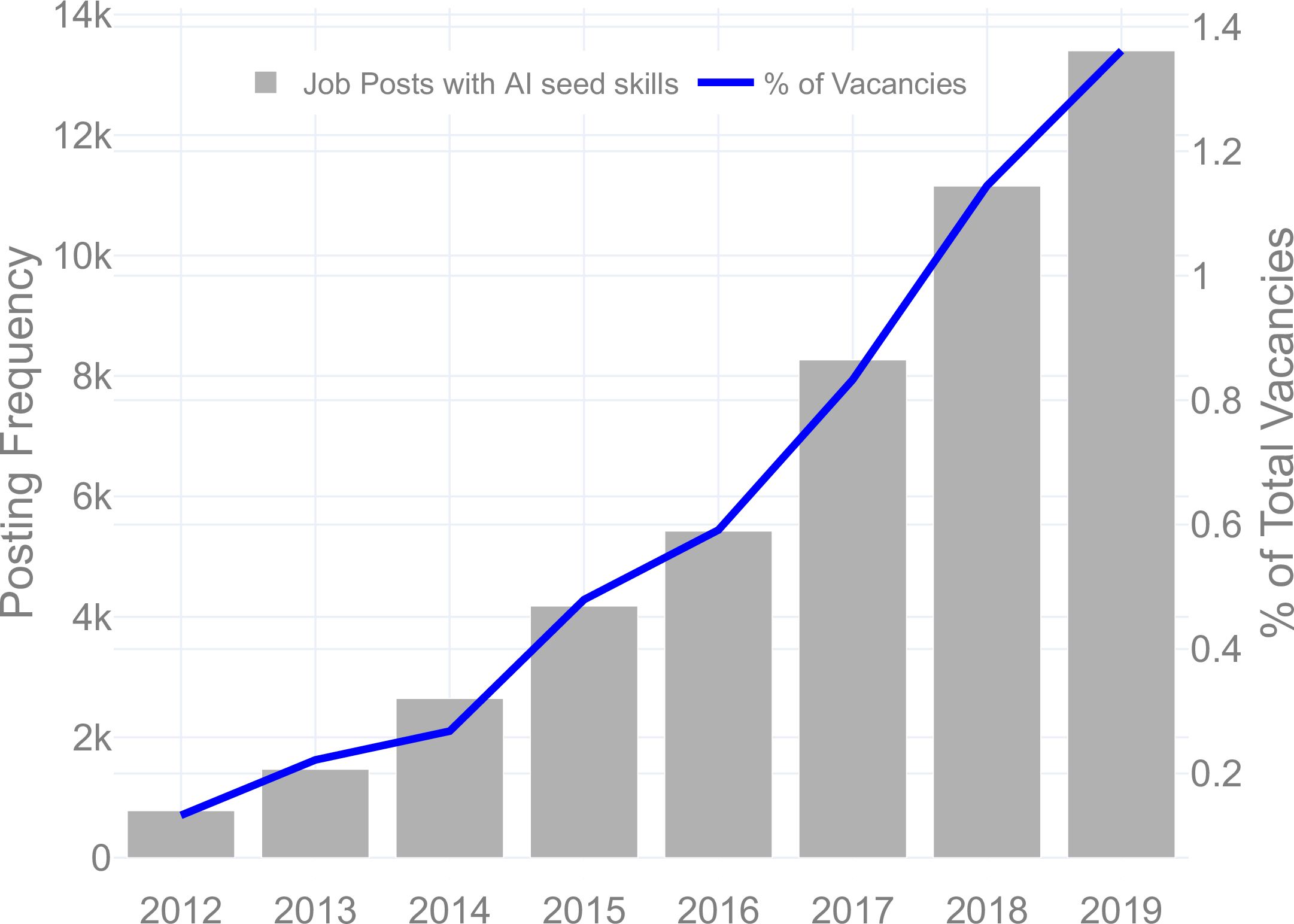

Supplement: S7 Fig — The percentage of vacancies in Australia that contain these five AI seed skills. (TIFF) [file pone.0254722.s008.tiff]
